# A Stat1 bound enhancer promotes Nampt expression and function within tumor associated macrophages

**DOI:** 10.1038/s41467-021-22923-5

**Published:** 2021-05-11

**Authors:** Thomas B. Huffaker, H. Atakan Ekiz, Cindy Barba, Soh-Hyun Lee, Marah C. Runtsch, Morgan C. Nelson, Kaylyn M. Bauer, William W. Tang, Timothy L. Mosbruger, James E. Cox, June L. Round, Warren P. Voth, Ryan M. O’Connell

**Affiliations:** 1 Division of Microbiology and Immunology, Department of Pathology, University of Utah, Salt Lake City, UT USA; 2 Huntsman Cancer Institute, University of Utah, Salt Lake City, UT USA; 3 Department of Biochemistry, University of Utah, Salt Lake City, UT USA; 4 Metabolomics Core Research Facility, University of Utah, Salt Lake City, UT USA

**Keywords:** Immunology, Gene regulation in immune cells, Innate immunity, Tumour immunology

## Abstract

Tumor associated macrophage responses are regulated by distinct metabolic states that affect their function. However, the ability of specific signals in the local tumor microenvironment to program macrophage metabolism remains under investigation. Here, we identify NAMPT, the rate limiting enzyme in NAD salvage synthesis, as a target of STAT1 during cellular activation by interferon gamma, an important driver of macrophage polarization and antitumor responses. We demonstrate that STAT1 occupies a conserved element within the first intron of *Nampt*, termed Nampt-Regulatory Element-1 (NRE1). Through disruption of NRE1 or pharmacological inhibition, a subset of M1 genes is sensitive to NAMPT activity through its impact on glycolytic processes. scRNAseq is used to profile in vivo responses by NRE1-deficient, tumor-associated leukocytes in melanoma tumors through the creation of a unique mouse strain. Reduced *Nampt* and inflammatory gene expression are present in specific myeloid and APC populations; moreover, targeted ablation of NRE1 in macrophage lineages results in greater tumor burden. Finally, elevated *NAMPT* expression correlates with IFNγ responses and melanoma patient survival. This study identifies IFN and STAT1-inducible *Nampt* as an important factor that shapes the metabolic program and function of tumor associated macrophages.

## Abstract (web summary)

IFNy can polarize macrophages in the tumour microenvironment to an inflammatory state and thereby contributes to their anti-tumour function. Here the authors show an underlying mechanism in this process is IFNy-driven STAT1 occupancy and activation of NRE1, a regulatory region within the NAMPT gene, thereby implicating inducible NAD salvage synthesis in TAM functions.

## Introduction

The tumor microenvironment (TME) is exceedingly complex, even within the narrow scope of tumor-associated macrophage (TAM) behavior. In addition to canonical immune activation pathways, manipulation and reprogramming of macrophage metabolic networks by other immune cells or the tumor also occurs, and these multifaceted changes influence both anti- and pro-tumor phenotypes displayed by TAMs. Recently, key factors that control macrophage metabolic states have begun to be identified, and are starting to shed light on these connections^1–4^. Importantly, it is becoming clear that this dynamic interplay between macrophage metabolism and inflammatory signaling pathways is central to our understanding of effective cancer therapies.

Interferon gamma (IFNγ) is a hallmark cytokine produced by tumor infiltrating lymphocytes, such as NK and T cells, and its activation of TAMs leads to tumor inhibition through multiple mechanisms^5^. Increased cytotoxicity, inhibition of angiogenesis, and reversal of immunosuppressive myeloid functions have all been classified as IFNγ dependent antitumor activities^6–8^. Nevertheless, the TME is commonly able to sculpt myeloid cell functions toward pro-tumorigenic programs, and this often involves modulation of IFNγ-mediated functions^5^. Thus, myeloid cells within the TME can exist in a spectrum ranging from tumoricidal to pro-tumorigenic. Recent data have also begun to link IFNγ to changes in macrophage metabolism. One study indicated that IFNγ targets mTOR and MNK kinases to regulate translation initiation via eIF4E^1^. Further, there is evidence that IFNs can also promote aerobic glycolysis, albeit through a mechanism that is incompletely understood^3,9^. IFNγ and other pro-inflammatory signals reprogram macrophages to upregulate glycolysis, and this appears to be critical for many aspects of the inflammatory phenotype^10–12^. Despite this important progress, less is known about how IFNs might regulate macrophage metabolism through transcription-dependent mechanisms.

Nicotinamide phosphoribosyl transferase (NAMPT) is the essential rate limiting enzyme that initiates the mammalian NAD salvage synthesis pathway. Its dysregulated expression is observed in many human diseases, including acute lung injury, diabetes, and rheumatoid arthritis^13^. Originally discovered as pre-B-cell colony-enhancing factor^14^, NAMPT was later shown to promote immune cell development, survival, and function^15–17^. The NAMPT salvage pathway can be manipulated pharmacologically to reduce NAD and thereby limit tumor cell metabolism^18,19^, or to enhance NAD levels for neuroprotection^20^. *Nampt* is ubiquitously expressed, yet has also been shown to increase to higher levels in response to inflammatory stimuli^21,22^. However, the mechanisms surrounding this upregulation, and the biological consequences of this induction in the TME, are still being studied.

Despite being essential for cell viability, tissue-specific knockout and inhibition strategies have been applied to characterize NAMPT function. For instance, recent work using a driver for deletion of *Nampt* coding sequences specifically in myeloid cells, while altering immune function of some cell types, resulted in only modest reduction of NAMPT protein levels^23^. This probably reflects incomplete ablation due to the conflict between full deletion and cell survival. Thus far, no current model exists to study the relevance of inducible *Nampt* gene expression without altering baseline and essential functions. Such a model would reveal additional insight into the role of *Nampt* expression in macrophages and other immune cells during antitumor immunity.

Here, we searched for previously unknown mechanisms through which IFNγ signaling regulates TAM metabolism. By performing RNA-Seq on TAMs from tumors grown in WT vs. IFNγ receptor (IFNγR)-deficient mice, and through comparison with bone marrow-derived macrophages (BMMs) stimulated with LPS/IFNγ, we identify a subset of metabolic genes, including *Nampt*, as being strongly upregulated through an IFN-dependent process. We describe a mechanism through which *Nampt* is induced in both mouse and human myeloid cells, through direct STAT1 occupancy in vivo of a previously unrecognized conserved element we have identified in the first intron of *Nampt*, termed Nampt Regulatory Element-1 (NRE1), in contrast to other potential motifs in the *Nampt* promoter. Through deletion of NRE1 as well as pharmacological inhibition of NAMPT, we demonstrate that IFNs induce *Nampt* through NRE1, a process critical for aerobic glycolysis and expression of a subset of inflammatory genes. Using single-cell RNA-Seq, we analyze tumor infiltrating immune cells from mice specifically lacking the NRE1 region in hematopoietic cells and find inflammatory and metabolic defects in monocyte and APC populations, and defective control of tumor growth. Upon targeted deletion of this regulatory region of *Nampt* in specific myeloid cells including TAMs, we observe significant loss of the ability to control melanoma tumors. Finally, human clinical data from The Cancer Genome Atlas (TCGA) database reveals relationships between *NAMPT* expression, immune signatures of melanomas, and other tumors, inflammatory status, and patient survival. Overall, our study identifies STAT-dependent NAMPT induction as a mechanism by which IFNs regulate macrophage metabolic shifts and consequently their function, and expands our understanding of how IFNγ promotes the function of TAMs in the TME.

## Results

### *Nampt* is upregulated by IFNs in TAMs through Jak/Stat1 signaling

**Fig. 1:**
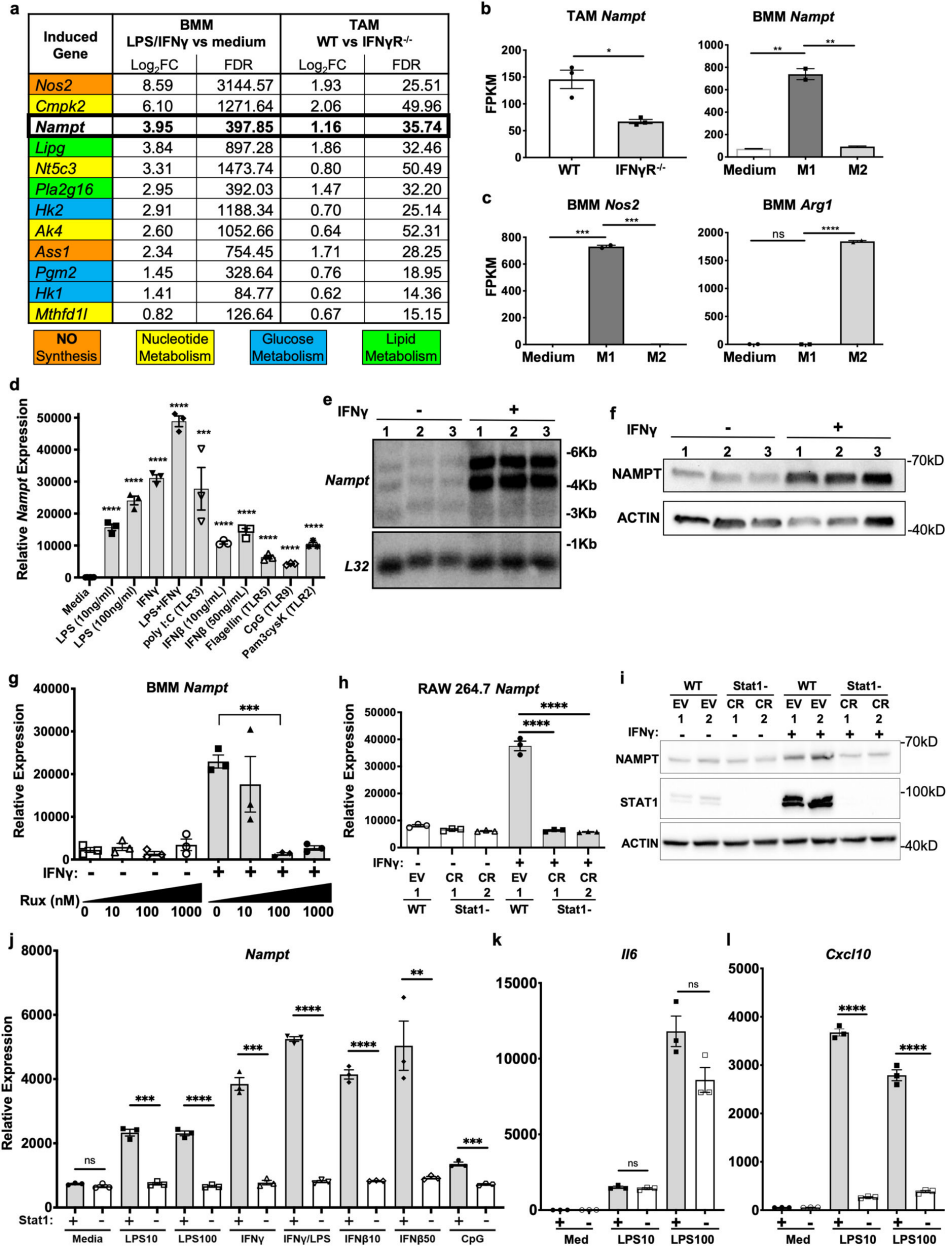
*Nampt* is upregulated by IFNs and TLR ligands in M1 macrophages and TAMs. **a** Table showing top 12 genes from BMM LPS/IFNγ vs. media treated cells and WT vs. IFNγR^−/−^ TAMs that were significantly upregulated by this M1 stimulation or were up significantly in WT TAMs. Log_2_FC log (base 2) of the expression fold change, FDR false discovery rate. **b** FPKM of *Nampt* from sequencing experiments described in **a**. Flow cytometric sort purification gating strategy provided in Supplementary Fig. 1A. **c** FPKM of *Nos2* and *Arg1* from M1 stimulated (LPS/IFNγ) vs. M2 stimulated (IL-4) BMMs. Error bars represent SEM for *N* = 3, or range for *N* = 2 independent RNA-seq samples. **d** qPCR of *Nampt* in BMMs stimulated for 6 h with the indicated PAMPs. **e** Northern blot analysis of *Nampt* from IFNγ stimulated BMMs made from B6 WT mice. Size markers are in kB. **f** Western blot of NAMPT from BMMs treated with IFNγ for 24 h. Size markers are in kDal. **g** Expression of *Nampt* in BMMs treated with Ruxolitinib at the indicated concentrations with and without IFNγ stimulation. **h** *Nampt* expression in RAW 264.7 cells in which STAT1 expression was deleted via CRISPR/Cas9 targeting of Stat1 (Stat1 CR) or non-targeting controls (WT EV). **i** Western blot of NAMPT, STAT1, and ACTIN from RAW 264.7 cell lines described in **c**. **j** *Nampt*, **k** *IL6*, and **l** *CXCL10* mRNA expression of cells described in **h** that were stimulated with the indicated PAMPs for 6 h. WT EV *STAT1*+ non-targeting controls or *STAT1*− CR deletions via CRISPR/Cas9 targeting are designated on the *x*-axes by Stat1+ or −, respectively. Data are representative of at least three independent experiments, except for RNA-seq results in **a**–**d**, **j** (two independent experiments). Error bars represent SEM for *N* = 3 biologically independent samples. *p* values were determined by a two-tailed unpaired *t*-test: *p < 0.05, **p < 0.005, ***p < 0.0005, ****p < 0.00005. EV non-targeting CRISPR gRNA control. Uncropped images provided in Source Data file in Supplementary information.

To identify metabolic regulators of macrophage function during the response to IFNγ within the TME, we FACS-isolated TAMs from WT and IFNγR^−/−^ mice challenged with syngeneic B16f10 melanoma tumors (Supplementary Fig. 1A), performed RNA sequencing, then compared metabolic genes from the KEGG pathway dataset to data generated from primary mouse BMMs stimulated in vitro with LPS/IFNγ. Twelve metabolic genes, including *Nampt*, were significantly upregulated, both within TAMs in the WT TME as well as by LPS/IFNγ stimulation in vitro (Fig. 1a). *Nampt* upregulation required intact IFNγR signaling in TAMs, and only stimulation with LPS/IFNγ (“M1” conditions), but not IL-4 (“M2” conditions), resulted in *Nampt* upregulation in BMMs (Fig. 1b). As expected, the stimulation of BMMs with LPS/IFNγ or IL-4 resulted in expression of either the pro-inflammatory M1 marker *Nos2* (Nitric oxide synthase 2), or the anti-inflammatory M2 marker *Arg1* (Arginase 1) by these populations, respectively (Fig. 1c). *Nampt* mRNA was induced in response to several inflammatory stimuli in primary BMMs, in particular those known to induce IFN autocrine/paracrine signaling (Fig. 1d). We noted the increased expression of *Nampt* at both the mRNA and protein levels using Northern and Western blots in BMMs stimulated with IFNγ alone (Fig. 1e, f).

Next, we wanted to determine the upstream signaling pathways required for the inducible regulation of *Nampt* in response to IFNγ. Upregulation of *Nampt* in response to IFNγ in primary BMMs occurred within an hour of stimulation and was sustained for at least 48 h (Supplementary Fig. 1B), suggesting the involvement of a transcription factor with the ability to rapidly upregulate the production of *Nampt*. Jak/Stat signaling is a known signaling pathway downstream of IFNγ that we hypothesized would be involved in regulating *Nampt* expression. To test this, we first utilized the Jak1/Jak2 inhibitor Ruxolitinib and observed a dose-dependent effect of this drug on *Nampt* expression in IFNγ-stimulated BMMs (Fig. 1g). We then deleted the STAT1 protein from RAW 264.7 cells, a mouse macrophage cell line, with CRISPR/Cas9 technology and found this transcription factor is required for the IFNγ-inducible increase in *Nampt* mRNA and protein expression, but not for the baseline expression of *Nampt* (Figs. 1h, i). These Stat1-dependent effects on *Nampt* were in parallel to the canonical Stat1 target CXCL10 (Supplementary Fig. 1C, D). Further, increased expression of *Nampt* in response to LPS, IFNγ, the LPS/IFNγ combination, and IFNβ also required intact STAT1 signaling (Fig. 1j). As controls, the induction of *Il6* was not significantly impacted by loss of STAT1 (Fig. 1k). The pro-inflammatory cytokine gene *Il6* is strongly induced by LPS in a manner dependent on the TLR4-NF-κB pathway^24^, but at a high LPS dose this induction does show some Stat1 dependence (Fig. 1k). Additionally, *CXCL10* activation, which does directly require STAT1, was strongly decreased (Fig. 1l). Together, these data indicate a role for the Jak/Stat pathway in regulating NAMPT expression, and the requirement for Stat1 in *Nampt* responses to a spectrum of inflammatory stimuli.

### NRE1 is an intronic STAT1 site regulating IFNγ-mediated *Nampt* induction

**Fig. 2:**
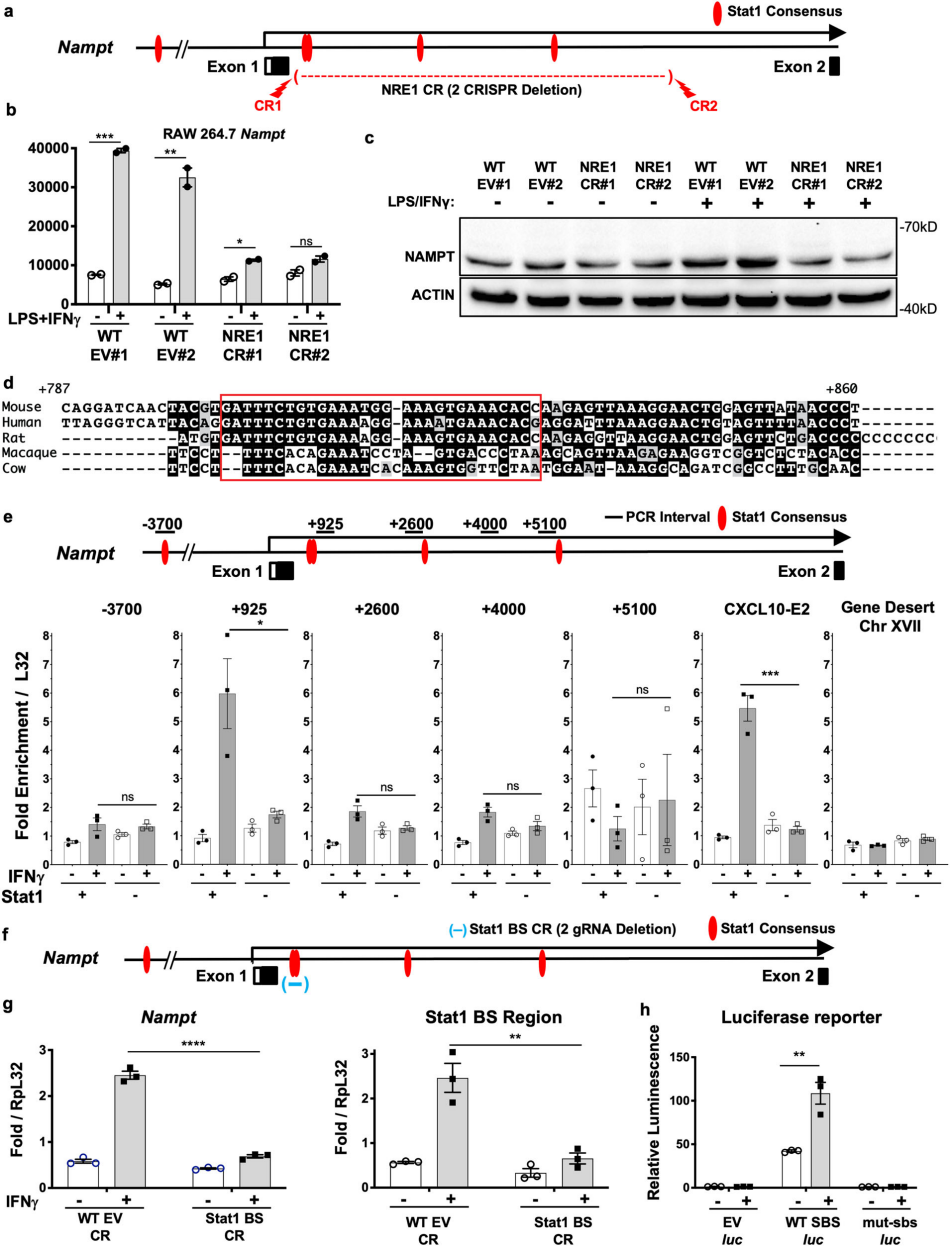
IFNγ inducible expression of *Nampt* in macrophages through NRE1. **a** Schematic of the first intron of *Nampt*, which contains the indicated predicted STAT1 binding sites, that we termed NRE1. CRISPR/Cas9-mediated deletion of this site was accomplished with the indicated constructs (CR1, CR2). **b** qPCR of *Nampt* expression in EV WT clones and NRE1 CR clones with and without LPS/IFNγ stimulation for 6 h. **c** Western blot of indicated cells treated with LPS/IFNγ for 24 h. Size markers are in kB. **d** Conservation of STAT1 binding site located within the first intron of *Nampt*. **e** Chromatin immunoprecipitation with anti-STAT1 antibody analyzed by qPCR of the indicated regions in RAW 264.7 Stat1 EV cells and Raw 264.7 Stat1 CR cells. **f** Schematic of the CRISPR mediated deletion of the STAT1 binding site (BS) located within NRE1 and the first intron of *Nampt*. **g** RT-qPCR expression measurement of RNA from *Nampt* mRNA or the STAT1 BS region of NRE1 from RAW 264.7 STAT1 BS CR cells and controls, with and without 6-h stimulation with IFNγ. **h** *Oplophorus* (NanoLuc) luciferase reporter constructs with no added sequence (EV), WT NRE fragment containing the STAT1 binding sites (SBS), or scrambled STAT1 binding sites (sbs-m) were transduced into RAW 264.7 cells treated with IFNγ or medium alone. Resulting luminescence was measured and normalized relative to a co-transduced *Photinus* luciferase positive control. For **b**, **e**, **g**, **h**, results presented are the average of *N* = 3 biological replicates, and are representative of at least three independent experiments. *p* values were determined by a two-tailed unpaired *t*-test: *p < 0.05, **p < 0.005, ***p < 0.0005, ****p < 0.00005. Error bars represent SEM. EV non-targeting CRISPR gRNA control. Uncropped images and raw scans provided in Source Data file in Supplementary information.

While we and others have noted a number of potential STAT1 DNA binding sites throughout the promoter region that might possibly account for the IFNγ inducible expression of *Nampt*^25^, such short sequence motifs also pervade the genome in the absence of any functional relevance. However, *bona fide* Stat1-regulated genes also possess elements with similar consensus motifs that are not immediately distinguishable from non-functional sequences. Thus, we undertook a more thorough analysis of STAT1 activity in programming *Nampt* IFNγ-dependent induction. Using JASPAR^26^ to scan all potential regulatory regions of *Nampt*, we identified several STAT1 motifs within its first intron. We further observed elevated transcript levels based on RNA-Seq reads mapping to the first intron of *Nampt* in primary BMMs stimulated with LPS/IFNγ (Supplementary Fig. 2A), consistent with enhancer activity. To determine if this intronic region was regulating *Nampt* expression by functioning as a bona fide transcriptional enhancer, we utilized CRISPR/Cas9 constructs carrying sgRNAs directed against either boundary of this region of *Nampt* in RAW 264.7 cells, which we have termed NRE1 (Fig. 2a). This method created clonal populations of cells with deletions and mutations of NRE1, which we verified by Sanger sequencing of genomic PCR products (Supplementary Fig. 2B). NRE1 deletion clones expressed normal baseline levels of *Nampt* but had a diminished capacity to upregulate *Nampt* following IFNγ/LPS stimulation at both the mRNA and protein levels (Fig. 2b, c). This is also seen under IFNγ only conditions (Supplementary Fig. 2C), with CRISPR/Cas9 deletion of *Stat1* serving as a control for inducibility of *Nampt*. Importantly, northern blot analysis detected no shift in the size of *Nampt* mRNA isoforms in the NRE1 CRISPR/Cas9 clones compared to control clones, indicating normal splicing of the mutant locus (Supplementary Fig. 2D). Correlating with the loss of IFNγ inducibility of *Nampt* in the absence of NRE1, NAD+ abundance in these cells as measured by liquid chromatography–mass spectrometry (LC-MS) was also significantly reduced (Supplementary Fig. 2E).

We next hypothesized that NRE1 contained a required binding site for STAT1 to program the inducible upregulation of *Nampt* during IFNγ stimulation. Analysis of the conservation of this region revealed dual adjacent STAT1 binding sites (SBS) common among multiple mammalian species, including humans (Fig. 2d). While there is not extensive homology extending throughout the 6kB NRE1 with any other area of the genome, well conserved minimal Stat1 binding motifs similar to these within NRE1 are readily identifiable in the regulatory regions of other IFN coordinately regulated genes. Chromatin immunoprecipitation (ChIP) of STAT1-associated DNA in RAW 264.7 macrophages revealed this region, located ~800 base pairs downstream of exon 1 of *Nampt*, had increased STAT1 occupancy in cells stimulated with IFNγ, which was dependent upon STAT1 expression (Fig. 2e). We saw only basal levels of STAT1 at the other putative SBS in NRE1 or the promoter region. Therefore, we designed a dual sgRNA CRISPR/Cas9 targeting construct (Stat1 BS CR) to delete the small (78 bp) region of NRE1 containing the dual STAT1 motifs (SBS), where correspondingly the highest increase in STAT1 binding was observed by ChIP (Fig. 2f). Cell clones with verified deletion and/or mutation of this region had defects in their capacity to induce *Nampt* in response to IFNγ (Fig. 2g). Cloning of this region into an ectopic luciferase reporter plasmid resulted in IFNγ responsive luciferase expression in RAW 264.7 macrophages, which was then abrogated by replacing the SBS located in this fragment with a scrambled sequence (Fig. 2h). These results demonstrate that NRE1 contains a SBS that contributes to the inducible upregulation of *Nampt* by IFNγ.

### NAMPT enzyme inhibition blunts IFNγ-mediated inflammatory responses

**Fig. 3:**
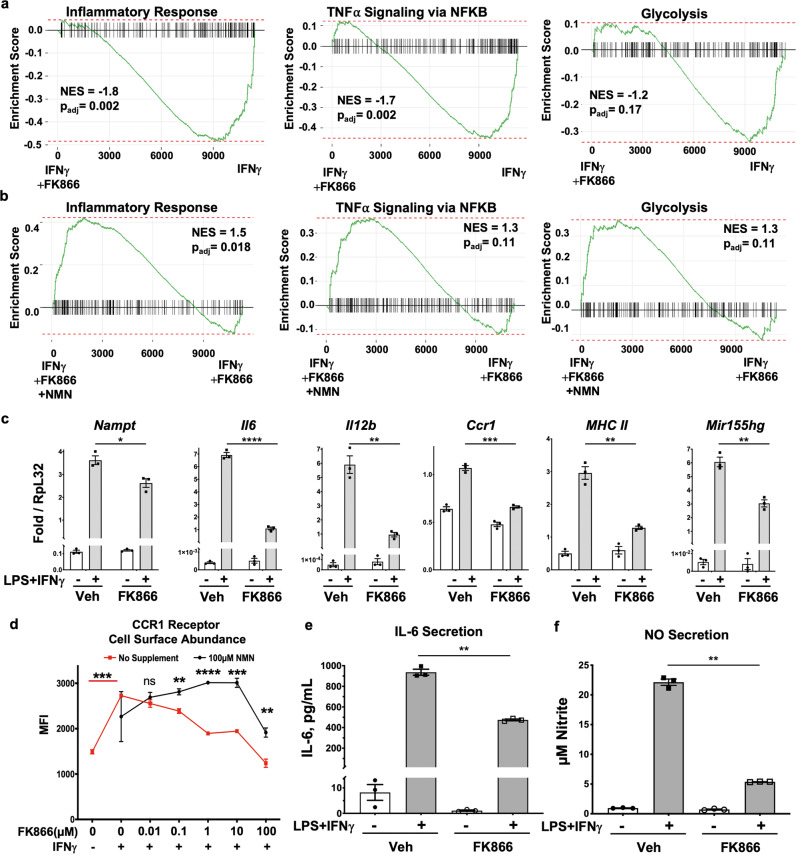
NAMPT is required for inflammatory expression programs during IFNγ responses. **a** RNA-seq was performed on primary BMMs in the presence of IFNγ, and/or FK866, and NMN. GSEA signatures for inflammatory and glycolysis pathways, showing the enrichment of genes whose IFNγ-dependent induction is lowered in the presence of the FK866 NAMPT inhibitor treated BMMs. **b** The same GSEA pathways showing rescue of FK866-dependent inhibition by NMN. For **a**, **b**, NES normalized enrichment score, *p*_adj_ adjusted *p* value: two-tailed, corrected for multiple comparisons using Benjamini–Hochberg method. **c** RT-qPCR expression of indicated inflammatory genes in the presence of FK866 under LPS/IFNγ stimulation. **d** Flow cytometry measurement of mean fluorescence intensity for CCR1 abundance on the cell surface in the presence of the indicated treatments. **e** IL-6 cytokine secretion into culture medium after 24 h treatment with indicated conditions as measured by ELISA, and quantified using purified standard dilutions. **f** NO secretion measured by Griess assay of nitrite levels in culture medium in the same conditions as in **e**. For **c**–**f**, results presented are the average of *N* = 3 biological replicates, and are representative of at least three independent experiments. *p* values were determined by a two-tailed unpaired *t*-test: *p < 0.05, **p < 0.005, ***p < 0.0005, ****p < 0.00005. Error bars represent SEM.

We explored the function of NAMPT following its induction by IFNs in macrophages. RNA-Seq was performed on BMMs treated with IFNγ and the investigational drug FK866, a NAMPT inhibitor that has been shown to block production of nicotinamide mononucleotide (NMN), the immediate precursor to NAD in the salvage pathway^18,19^. Results indicate that such inhibition of NAMPT causes decreased expression of inflammatory response, TNFα signaling, and also glycolytic pathways that are normally induced in response to IFNγ, as determined using GSEA (Fig. 3A). Defective induction of these pathways was rescued by further inclusion of NMN, the immediate product of NAMPT, to the otherwise identical experimental setting (Fig. 3B). The IFNγ response gene set itself is also altered markedly in the presence of FK866-inhibited NAMPT (Supplementary Fig. 3A vs. B). Addition of NMN partially restores induction of the IFNγ response gene set to that seen in IFNγ treatment alone (Supplementary Fig. 3C), suggesting that many aspects of the IFNγ program are impacted by NAD produced from NAMPT, including upregulation of glycolysis.

In order to analyze the dependence of NAMPT and NAD-dependent processes on M1 macrophage activation more quantitatively, we evaluated *Il6*, *Il12b* (IL-12p40), and several genes encoding other inflammatory factors in their responses to LPS/IFNγ. We observed that their induction was reduced upon treatment with FK866 as measured by RT-qPCR, consistent with a role for NAMPT-produced NAD during responses by classically activated M1 macrophages (Fig. 3c). Among genes whose inducible expression was affected by FK866 was *Ccr1*, encoding a cell surface receptor important for immune cell recruitment to inflammatory sites^27,28^.

Using flow cytometry, we found that CCR1 cell surface abundance levels were also reduced by FK866 in a dose-dependent manner (Fig. 3d). However, normal CCR1 levels could be restored by exogenous addition of NMN, even in the presence of increasing amounts of inhibitor. This effect was specific, because cell surface expression of the related receptor CCR2 was unaffected by these treatments in the same samples (Supplementary Fig. 3D). ELISA analysis of the secreted pro-inflammatory cytokine IL-6 from BMMs also showed blunted induction by LPS/IFNγ in the presence of inhibited NAMPT (Fig. 3e), while induced secretion of nitric oxide, a hallmark of macrophage inflammatory response, was also strongly reduced (Fig. 3f). To verify that the dosages of FK866 used in these assays were effective at reducing the IFNγ stimulated high levels of NAD, but were also not causing toxicity effects, LC-MS-based measurements of NAD+ under increasing doses of FK866 indicated that non-toxic concentrations of FK866 were able to abrogate IFNγ-induced NAMPT levels of activity (Supplementary Fig. 3E, F).

### NRE1 deletion in BMMs reduces an IFNγ induced *Nampt*-dependent program

**Fig. 4:**
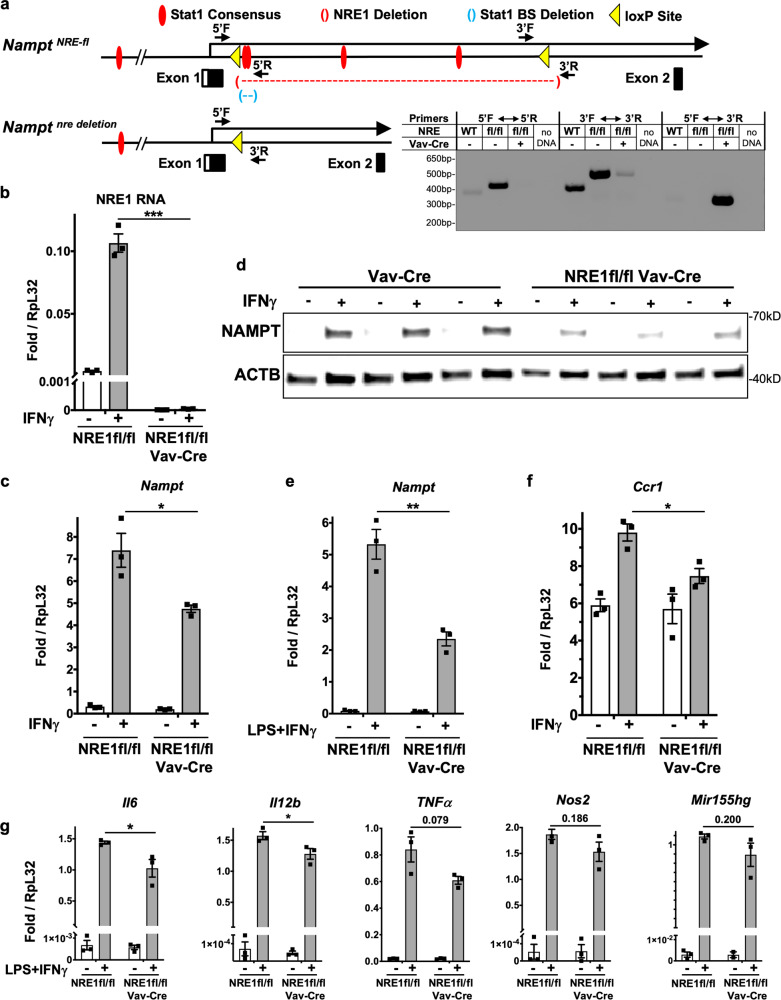
NRE1 deletion reduces NAMPT induction and LPS/IFNγ inflammatory responses. **a** NRE1 Cre-lox knockout scheme: diagram indicating inserted loxP sites bounding NRE1 relative to STAT1 consensus sites, CRISPR deletion alleles, and loxP detection primers, in both the floxed and deleted alleles. Representative agarose gel showing PCR products from macrophages using the indicated primers for the presence of each loxP site relative to WT (non-floxed) NRE1, and generation of a deletion junction product from Vav-Cre NRE1 fl/fl (NRE1-KO) primary bone marrow macrophages. Size markers are in bp. **b** RT-qPCR from the indicated macrophage RNAs shows the absence of NRE1 signal in the deletion. **c** Mature *Nampt* mRNA expression measured by RT-qPCR from WT and NRE1-KO macrophage RNAs treated with IFNγ. **d** Western analysis of multiple IFNγ or untreated macrophage cultures from mice of the indicated genotypes showing lowered inducibility of NAMPT protein relative to ACTIN controls. **e** Mature *Nampt* mRNA expression in the presence of LPS/IFNγ is blunted in the absence of NRE1 in Vav-Cre NRE1 fl/fl (NRE1-KO) macrophages. **f** Ccr1 mRNA expression measured by RT-qPCR from WT and NRE1-KO macrophage RNAs treated with IFNγ. **g** Inflammatory and glycolytic pathway genes’ mRNA expression measured by RT-qPCR from WT and NRE1-KO macrophage RNAs treated with IFNγ and LPS. For **b**, **c**, **e**–**g**, results presented are the average of *N* = 3 biological replicates, and are representative of at least three independent experiments. *p* values were determined by a two-tailed unpaired *t*-test: *p < 0.05, **p < 0.005, ***p < 0.0005, ****p < 0.00005. Error bars represent SEM. Uncropped images and raw scans provided in Source Data file in Supplementary information.

To further investigate STAT1-dependent activation through the NRE1 during primary macrophage responses to IFNγ, we created mice with a floxed NRE1 sequence. This allele allows the in vivo decoupling of IFN-dependent regulation of *Nampt* from the global STAT-driven inflammatory program in any cell type. This line was then crossed to Vav-Cre mice to delete NRE1 in hematopoietic cells (Fig. 4a). Appropriate insertion of the loxP sites and tissue-specific deletion of NRE1 was confirmed by PCR and RT-qPCR (Fig. 4a, b). Bone marrow was collected from these and control mice and used to generate BMMs in vitro. Upon stimulating these cells with IFNγ, we found that induction of *Nampt* was reduced at the mRNA and protein levels (Fig. 4c, d), and the ability to upregulate NAD+ production in response to IFNγ was likewise defective (Supplementary Fig. 4A). This requirement for NRE1 was also observed in M1 activation conditions with LPS and IFNγ (Fig. 4e). Further, we found that treatment of NRE1-deficient BMMs with IFNγ or LPS/IFNγ resulted in significantly reduced expression of *Ccr1, Il6, IL12b*, with trending reductions in *miR155* and other pro-inflammatory factor genes (Fig. 4f, g), indicating that *Nampt* activity may affect subsets of the M1 program but not all aspects of inflammation in macrophages. There may be other as yet uncharacterized distal Stat1-dependent activation elements for *Nampt*, and also these critical inflammatory response genes are controlled by diverse signaling pathways in addition to inducible *Nampt*/NAD-dependent activation. Overall, however, these experiments reveal a role for NRE1 in regulating *Nampt* expression and subsequent inflammatory gene expression during the IFN response in primary macrophages.

### Induced NAMPT/NAD drives LPS/IFNγ-dependent glycolysis and TCA cycle

**Fig. 5:**
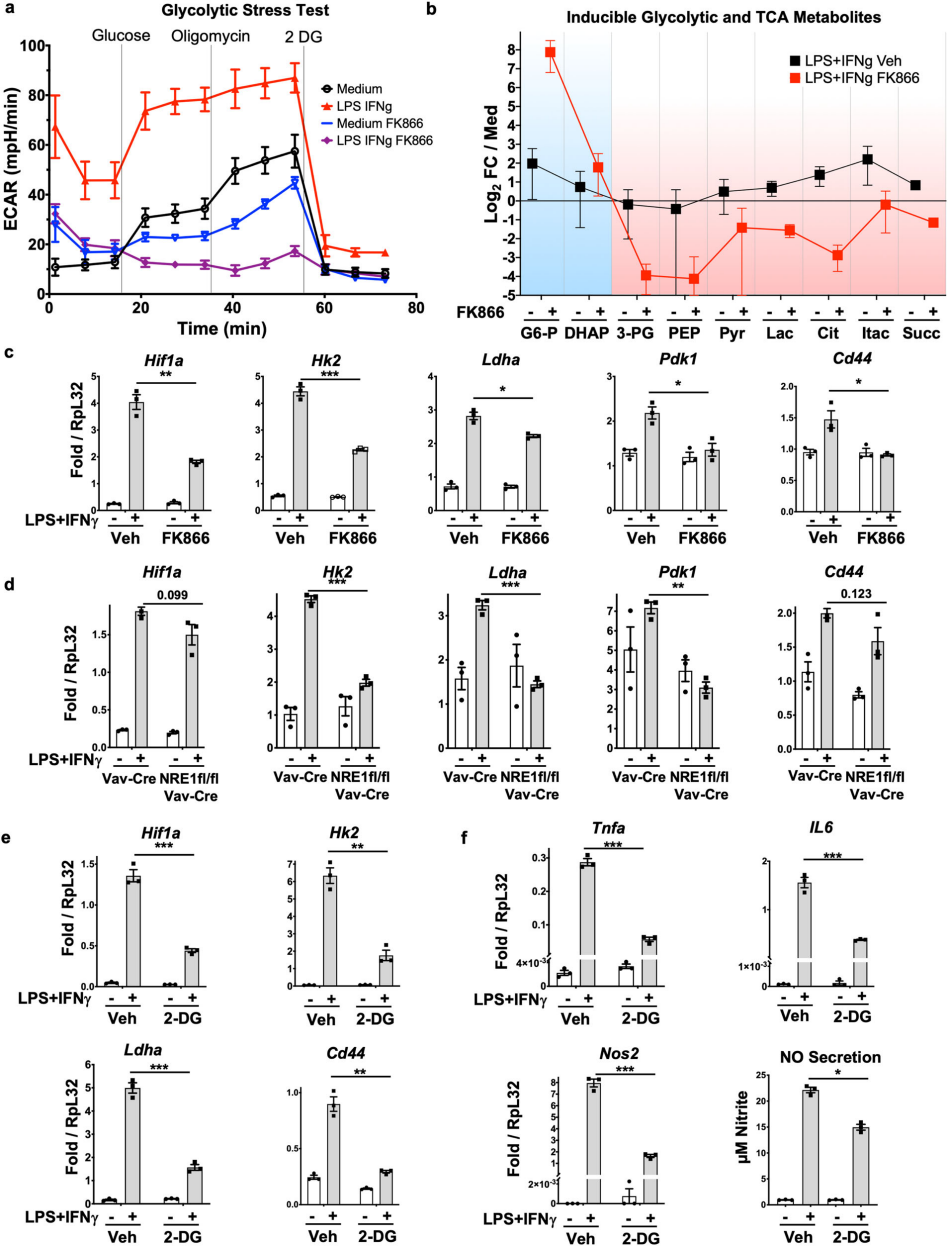
NAMPT enzymatic function is required for LPS/IFNγ-induced glycolysis at GAPDH. **a** Glycolytic stress test showing secreted lactate as measured by ECAR (extracellular acidification rate) for BMMs treated with combinations of LPS/IFNγ and FK866. Error bars represent SEM for *N* = 10 biological replicates. **b** Glycolytic and TCA cycle intermediate metabolites in BMMs treated with combinations of LPS/IFNγ and FK866. Values on *Y*-axis are Log_2_ ratios of LPS/IFNγ induced metabolite levels normalized as Fold Change vs. non-inducing medium only (black), or medium with FK866 only (red). Blue background indicates those steps prior to the first NAD requiring GAPDH step, and red background indicates those steps after GAPDH. *X*-axis categories are metabolites in pathway order as in Supplementary Fig. 5A^86^. Statistical significance for the effects of FK866 on induction are reported in Supplementary Fig. 5B. **c** Expression of individual glycolytic gene set members as measured by RT-qPCR from the indicated macrophage RNAs when treated with combinations of LPS/IFNγ and FK866, showing reduction of inducible expression in the presence of FK866-inhibited NAMPT. **d** Expression of the same glycolytic gene set members as in **c** from Vav-Cre alone or Vav-Cre NRE1 fl/fl (NRE1-KO) macrophage RNAs treated with LPS/IFNγ, showing loss of induction in the absence of the NRE1. **e** Inducible glycolytic and **f** inflammatory gene expression is lost in the presence of the glycolysis inhibitor 2-Deoxy-D-Glucose. Results presented are the average of *N* = 3 biological replicates. Except for **a**, **b**, **e**, **f**, data are representative of at least three independent experiments. For **b**–**f**, results presented are the average of *N* = 3 biological replicates. *p* values were determined by a two-tailed unpaired *t*-test: *p < 0.05, **p < 0.005, ***p < 0.0005, ****p < 0.00005. Error bars represent SEM.

The interplay between induction of *Nampt*, glycolysis, and classical M1 mode activation was investigated by further characterizing the specific requirements in glycolysis for *Nampt* activity during LPS/IFNγ induction. Using lactate production measured by ECAR (extracellular acidification rate) as an assessment of glycolysis activity, we found that the strong increase in glycolytic capacity typical of LPS/IFNγ activated BMMs is markedly reduced in the presence of FK866, with glycolytic rates and capacity nearly absent (Fig. 5a).

In order to more precisely characterize the enzymatic steps in glucose utilization and subsequent energy metabolism that are affected by loss of inducible NAMPT activity, we performed gas chromatography–mass spectrometry (GC-MS) based metabolomics in BMMs treated with combinations of LPS/IFNγ and FK866, analyzing glycolytic and TCA cycle intermediates (Fig. 5b and Supplementary Fig. 5A, B). Metabolite levels measured in the presence of LPS/IFNγ only or LPS/IFNγ and FK866 together were normalized to those in medium only, or with FK866 only groups, respectively (Fig. 5b). Detected glucose catabolic intermediates produced upstream of the GAPDH enzyme-catalyzed step of glycolysis, including G-6-P and DHAP (see Supplementary Fig. 5A), accumulated to higher levels in M1-activated BMMs treated with FK866 relative to normal medium. We interpret this buildup as due to GAPDH being the earliest enzyme in this pathway with a requirement for NAD, and that FK866-dependent reduction of NAD inhibits the normally efficient substrate conversion observed in activated control cells. However, those intermediate products downstream of GAPDH, including pyruvate and intracellular lactate, failed to accumulate to normal levels following inhibition of NAMPT. This pattern of blunted metabolite levels in M1-activated BMMs was also true for TCA cycle intermediates and products in the absence of normal NAMPT activity, at steps where NAD is additionally required. These metabolites include citrate, succinate, a molecule used for innate immune signaling^29^, and also itaconate, the product of IRG1-catalyzed conversion of the TCA intermediate *cis*-aconitate, and which has been implicated in regulating macrophage inflammatory ROS production by inhibiting subsequent steps in the TCA cycle^30^ (see Supplementary Fig. 5A). Together, these results indicate an important role for NAMPT production of NAD during glycolysis in IFNγ-activated macrophages.

We next needed to verify that these metabolomic results indicating defects throughout respiration as well as glycolysis were also evidenced by overall metabolic phenotype consequences. We additionally sought to test whether solely IFNγ-induced metabolic reprogramming was similarly reliant on the same NAMPT-dependent production of NAD as was found using both LPS and IFNγ. Thus, we subjected IFNγ treated cells to oxygen consumption rate (OCR) analysis using mitochondrial stress (MST) tests. Our data indicate that IFNγ activation of BMMs induces increased OCR during mitochondrial ATP production, which is also dependent on full NAMPT activity (Supplementary Fig. 5D), in that this increase is dose-responsive to FK866 inhibition. Activation using LPS/IFNγ applied to WT BMMs in the presence of FK866 also showed impaired induction of glycolytic pathway genes assayed individually by RT-qPCR (Fig. 5c). LPS/IFNγ treatment of BMMs bearing either floxed NRE1 alone (control) or Vav-Cre deleted homozygous NRE1 knockout alleles demonstrated a requirement for inducible *Nampt* for increased expression of glycolysis genes (Fig. 5d). To compare the abrogation of these responses by FK866 to those previously characterized during direct inhibition of glycolysis per se, we also measured gene expression following LPS/IFNγ activation of WT BMMs in the presence of the hexokinase inhibitor 2-Deoxy-glucose (2-DG)^31,32^. We observed reductions in glycolytic pathway gene activation (Fig. 5e) in addition to inflammatory factors induced by LPS/IFNγ (Fig. 5f). These results mimicked the effects of FK866, and confirmed the role of Nampt-supported glycolysis in promoting inflammatory gene expression in macrophages.

### Stat1-induced *Nampt* supports myeloid function in a mouse melanoma model

**Fig. 6:**
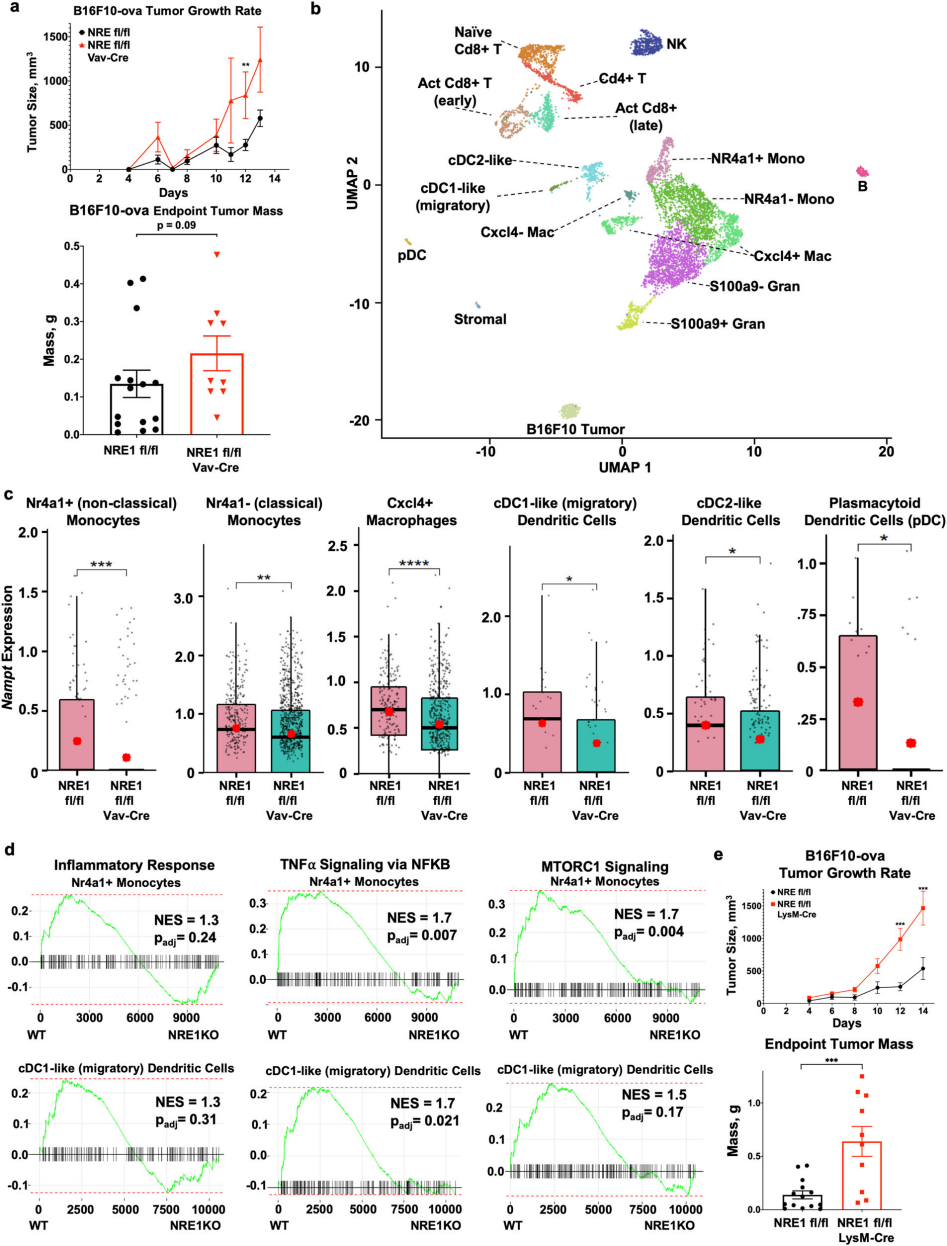
STAT1-inducible *Nampt* in the hematopoietic lineage during B16F10 melanoma. **a** B16F10-ova melanoma cell line flank tumor size (see “Methods”) growth rate and endpoint mass in WT or Vav-Cre NRE1 fl/fl (NRE1-KO) mice. **b** scRNAseq cell cluster analysis using UMAP plots for CD45+ cells isolated from B16F10-ova tumors in WT or NRE1 KO mice. Flow cytometric sort purification gating strategy provided in Supplementary Fig. 6, and Source Data file in Supplementary information. **c** *Nampt* expression in selected cell types from scRNAseq from WT or NRE1 KO mice. Box-and-whiskers plots show the distribution of gene expression in individual cells, and the red dot denotes average expression across the cells within the sample. Crossbars, boxes, and whiskers represent median, interquartile range, and total range of Nampt expression, respectively. **d** Pro-inflammatory and metabolic GSEA signatures in indicated cell types from scRNAseq, with positive enrichment of gene sets in wild-type on the left of each plot relative to NRE1 KO on the right. NES normalized enrichment score, *p*_adj_ adjusted *p* value: two-tailed, corrected for multiple comparisons using Benjamini–Hochberg method. **e** B16F10-ova flank tumor size growth rate and endpoint mass in WT or LysM-Cre NRE1 fl/fl (NRE1-KO) mice. Except for **b**–**d**, data are representative of three independent experiments. Results presented are the average of at least four biological replicates. For **a**, results presented are the average of *N* = 9 or 15 biological replicates, for **e**, *N* = 10 or 15 replicates, and are representative of at least two independent experiments. *p* values were determined by a two-tailed unpaired *t*-test: *p < 0.05, **p < 0.005, ***p < 0.0005, ****p < 0.00005. Error bars represent SEM.

In order to address the role of inducible expression of NAMPT in an in vivo cancer setting, mice bearing the homozygous NRE1 floxed allele with the broad hematopoietic lineage-specific deletion driver Vav-Cre, or their NRE1 floxed littermates lacking Cre, were inoculated subcutaneously with B16F10-ova melanoma cells^33^ to form flank tumors. Mice with blunted inducibility of hematopoietic cell *Nampt* due to tissue-specific deletion of NRE1 had larger tumors (Fig. 6a). To evaluate the NRE1-dependent function of tumor infiltrating immune cells, and to identify which specific immune subsets are implicated in NRE1-dependent tumor control, CD45-positive cells were isolated from these tumors grown in WT and NRE1-KO mice by FACS, subjected to single-cell RNA sequencing (scRNAseq), then analyzed using the Seurat and in-house developed^34,35^ computational platforms. Figure 6b illustrates the identification of cell types from each genotype by multidimensional gene expression profiles using uniform manifold approximation and projection comparison plots^35,36^. Full descriptions of the marker genes used as classification criteria to assign clusters, including expression level, frequency, and significance are in Supplementary Data 1. A total of 18 single-cell clusters were identified and annotated by using transcriptome-wide similarities to known mouse immune cell subsets in the Immunological Genome Project (ImmGen) database^37^. Closely related cell populations were distinguished by using differential expression analyses leading to a variety of both lymphoid and myeloid cell subsets, including monocyte populations differing in Nr4a1 and similar functionally relevant genes (consistent with classical and nonclassical monocyte phenotypes), granulocyte clusters classified by S100a9 status, macrophages classified by levels of Cxcl4, and dendritic cell types designated based on expression of several marker genes including cytokines and chemokines, and their receptors. Tumors from both genotypes have a similar distribution of immune cell types, notably there are no major differences in abundance of monocyte, macrophage, or dendritic cell lineages, with modest variation in relative cell quantities of certain other cell types (Supplementary Fig. 6A, B). For most myeloid and APC cell populations, the absence of NRE1 results in reduced *Nampt* expression (Fig. 6c), consistent with in vitro results using BMMs from these mouse lines. Further analysis using GSEA in two of these cell types, the Nr4a1 positive monocytes (consistent with a nonclassical monocyte profile) and mature dendritic cells, shows significant differences in gene expression profiles between WT and NRE1-KO. WT samples are enriched in several pro-inflammatory and pro-glycolytic pathways relative to NRE1-KO (Fig. 6d). Other cells in which *Nampt* is altered also show partial enrichment in some pathways including the pro-glycolytic MTORC1 signaling gene set (Supplementary Fig. 6E). Measurement of *Nampt* expression levels in all other cell clusters did not show significant differences in abundance where *Nampt* could be detected in substantial numbers of cells (Supplementary Fig. 6H). Also, comprehensive expression analysis of all identified clusters from both WT and NRE1-KO samples using Gene Ontology (GO) enrichment for Biological Process terms was performed, and all results are presented in Supplementary Data 2 and Supplementary Data 3. Overall, the above data suggest a scheme in which IFNγ/STAT inducible levels of *Nampt* support required metabolic and inflammatory responses by myeloid and APC cell types to curb malignancy. These differences were observed in mice with NRE1 deletion broadly in the hematopoietic compartment, encompassing many cell types with both positive and negative functions in tumor immunity, as well as in different stages of immune development. For instance, macrophages with a spectrum of pro- and anti-inflammatory characteristics^38,39^ coexist in the TME. Consistent with our other findings on the importance of NRE1 in bone marrow macrophage *Nampt* responses to IFNγ, the scRNAseq results directed us to hypothesize that NRE1 in TAM regulates antitumor function.

To test this more directly, we constructed mouse lines bearing the homozygous NRE1 floxed allele with the macrophage-selective deletion driver LysM-Cre. These or their NRE1 floxed littermates lacking Cre were again inoculated with B16F10-ova cells as above. Figure 6e shows that mice with blunted IFNγ-dependent induction of macrophage *Nampt* were significantly defective in their ability to control melanoma growth, by both growth rate and final tumor mass. Flow cytometric analysis of macrophages in these tumors again showed similar proportions regardless of genotype (Supplementary Fig. 6F), suggesting that TAM function rather than abundance is of importance. The requirement for macrophage NRE1 in tumor control may apply to other malignancies as well, as similar increases in tumor burden were observed using MC-38-ova colon adenocarcinoma derived subcutaneous flank tumors in the LysM-Cre NRE1 flox deletion context (Supplementary Fig. 6G). These observations are consistent with *Nampt* NRE1 as a critical point of control in TAMs during their IFNγ/STAT-driven antitumor program.

### *NAMPT* expression correlates with the IFNγ response and human melanoma outcome

**Fig. 7:**
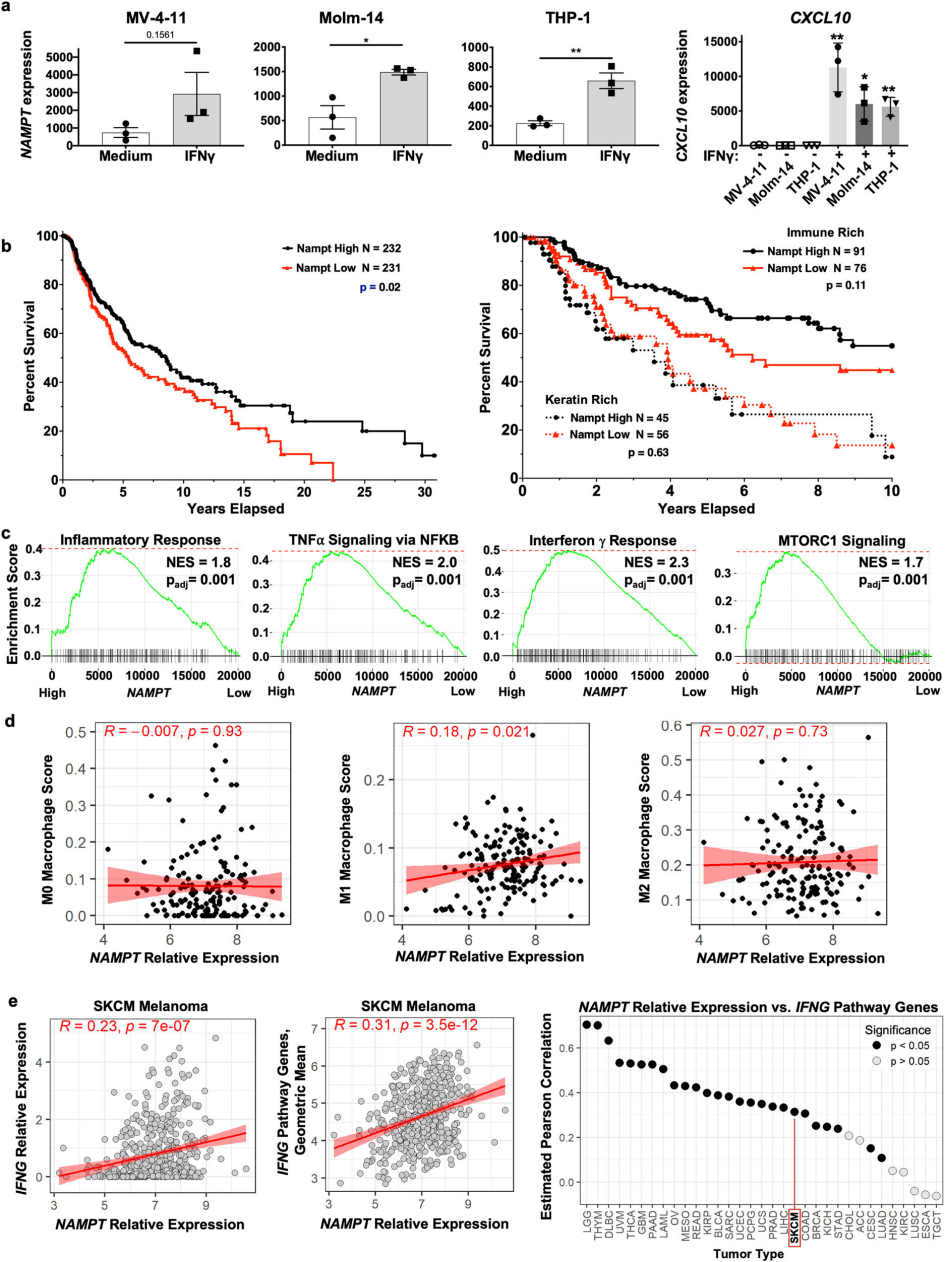
Correlation between human immune *NAMPT*, malignancy, inflammation, and outcome. **a** qPCR of *Nampt* from the indicated human myeloid cell lines that were stimulated for 6 h with IFNγ, with Hs *CXCL10* induction as a control for induction. Data are the average of *N* = 3 biological replicates, and are representative of at least three independent experiments. *p* values were determined by a two-tailed unpaired *t*-test: *p < 0.05, **p < 0.005, ***p < 0.0005, ****p < 0.00005. Error bars represent SEM. **b** Kaplan–Meier plots of TCGA full 30-year (left) or first 10-year (right) human melanoma patient survival data showing curves from median-divided *NAMPT* expression, that is, from patients with tumors expressing the highest vs. lowest 50% of *NAMPT* expression. The right plot is from data that were further stratified with respect to both the highest vs. lowest 10% of *NAMPT* expression levels and by classifying tumor samples cytologically as containing high vs. low immune infiltration. *p* values are reported within the figure panels for the difference between high and low *NAMPT* expression plots for either the keratin signature or immune signature-rich samples, and are from the log-rank (Mantel–Cox) test. **c** GSEA analysis for indicated pathways of gene expression data from TCGA melanoma transcriptomes in **b**. Gene rank order is determined by comparison of each gene’s expression between samples with high vs. low *NAMPT* levels. NES normalized enrichment score, *p*_adj_ adjusted *p* value: two-tailed, corrected for multiple comparisons using Benjamini–Hochberg method. **b**, **c** are based on data from a total of 463 patients. **d** Metagene signature analysis of NAMPT expression levels and macrophage subtypes in TCGA SKCM melanoma cohort, showing in immune rich tumors significant positive correlation of NAMPT abundance with inflammatory macrophage gene expression signatures (M1, middle). **e** Metagene signature analysis of *NAMPT* expression levels correlating with IFNγ induced responses from TCGA datasets for multiple tumor types in comparison to SKCM, where the *y*-axis shows Pearson correlation values. For **d**, **e**, *p* values are two-tailed, from tests for positive slopes of correlations between the two variables.

Because malignant tumors are often infiltrated by innate immune cells of myeloid origin, and their polarization characteristics and functional status in this IFNγ-rich environment influences disease progression, we investigated the role of immune cell *NAMPT* and its IFN/STAT induction in the human melanoma TME. We first assessed several human macrophage cell lines, which all responded to IFNγ by upregulating *NAMPT* expression (Fig. 7a), in parallel with the positive control IFNγ target *CXCL10*, leading us to investigate a role for this metabolic enzyme in regulating human tumor-associated immune responses.

Human melanoma tumor data from TCGA were analyzed for survival, degree of immune cell infiltration, and *NAMPT* expression level in tumor biopsies in the skin cutaneous melanoma (SKCM) collection. Long-term patient survival frequencies using the full available dataset were positively correlated with *NAMPT* expression, such that patients with higher than median expression showed statistically significant longer survival than those with below median expression in tumors, when analyzed using a Kaplan–Meier estimator (Fig. 7b). In order to assess the correlation of *NAMPT* expression in tumor-associated immune cells, these data were further stratified with respect to both *NAMPT* expression levels and tumors with high immune infiltration (assessed by immune-related gene expression signatures as reported previously^40^) vs. immune low (conversely high for the expression of tumor-related genes such as keratins). The 10-year survival data showed that the immune-low tumors were associated with a poor prognosis as expected, and that *NAMPT* expression level was not correlated with any difference in outcome. Conversely, while tumors scored as having a high level of infiltration by immune cells were similarly correlated with better disease outcomes, higher *NAMPT* expression was associated with a trend toward an increasingly improved prognosis within those immune rich tumors. Higher stringency stratification plots discriminated by the highest vs. lowest 10% *NAMPT* expression level cohorts in the patient population are shown in Supplementary Fig. 7A–C. While there are fewer data points in these curves, the differences in survival due to immune infiltration and high *NAMPT* expression are more significantly pronounced. By analyzing the gene expression profiles of these TCGA tumor samples using GSEA with respect to high vs. low *NAMPT* expression, we observed an increase in both pro-inflammatory (including IFNγ response genes), and pro-glycolytic signatures correlating with higher *NAMPT* expression (Fig. 7c). When we examined *NAMPT* expression relative to previously reported metagene signatures for intratumor macrophage subsets^34,41,42^, we noted a significant positive correlation between *NAMPT* levels and the M1 macrophage signatures within the immune-enriched melanoma, further supporting a functional role for IFNγ-induced TAM *NAMPT* in antitumor immunity (Fig. 7d). Furthermore, TCGA melanoma samples show a significant positive correlation between *NAMPT* and *IFNG* expression, and more broadly between *NAMPT* levels relative to the GSEA Hallmark IFNγ response gene set (Fig. 7e). Finally, when all malignancy types in the TCGA collection were analyzed for correlation of this IFNγ-induced metagene signature and *NAMPT* expression, many other cancers exhibited similar if not stronger positive correlations. Taken together, these clinical data suggest that *NAMPT* expression in the TME is coincident with increased immune infiltration and increased inflammatory status, and could support reduced disease.

## Discussion

In mice, whole body deletion of *Nampt* results in embryonic lethality, while studies utilizing tissue-specific Cre and floxed *Nampt* alleles have demonstrated the requirement of *Nampt* for survival and function of various cell types^15,43–45^. However, to date it has been challenging to address the relevance of upregulated NAMPT during disease states without disrupting basal NAMPT functions. In our study, we were able to gain critical insight into the regulation and function of inducible *Nampt* during macrophage activation by IFNγ through the identification and deletion of NRE1, which enabled us to begin to uncouple inducible from basal NAMPT function genetically. Our work points to an important role for increased *Nampt* in promotion of glycolysis and subsequently optimal inflammatory gene expression by TAM and other APC types.

*Nampt* is upregulated in a variety of human inflammatory diseases and cancers making it a gene of interest when considering improved therapeutic targets to treat such disorders^13,46,47^. However, the transcriptional regulation of *Nampt* within innate immune cells has been largely unstudied, with most work on *Nampt* transcriptional regulation limited to other cell types. In lung endothelial cells it was found that mechanical stress and exposure to LPS led to reduced promoter methylation and Stat5 dependent increased *Nampt* expression^48,49^. *Nampt* is also expressed by pancreatic β-cells to promote insulin secretion^50^ programmed by NCOA6, SREBP-1, and interaction with a sterol regulatory element within the promoter region of *Nampt*^51^. *Nampt* has also been shown to be transcriptionally regulated by Sirt1, for which NAMPT-dependent NAD is a coenzyme, by interaction with an E-box element within the *Nampt* promoter^52,53^.

We have newly identified and functionally characterized a conserved SBS located within the first intron of *Nampt*. Its occupancy and activation by STAT1 in response to inflammatory signals is the first description of a functional transcriptional regulatory element for *Nampt* in activated innate immune cells. Previous analyses have implicated *Nampt* intron 1 in regulation by other factors in some other cell types^52,53^, or have correlated IFNγ/STAT1 regulation with the presence of possible Stat1 binding motifs in other locations near *Nampt*^25^. While this motif analysis identified potential binding sites upstream of *Nampt*, in the absence of functional binding data, the relevance of these motifs is inconclusive. In this work, we observed little or no Stat1 occupancy in the proximal promoter region including these potential sites, but identified strong binding of Stat1 in vivo to chromatin in intron 1. Future work will be needed to understand how STAT1 binding to NRE1 coordinates transcription of *Nampt* in combination with some of the other elements and factors mentioned above under different contexts and in distinct immune cell types. This will be of importance because STAT1 plays a major role not only in the response to IFNs, but also to a variety of other cytokines and growth factors^54^. Thus, we expect further study of the STAT1 mediated regulation of *Nampt* to yield important insight into the potential roles of *Nampt* in innate immune cell inflammation and the development, differentiation, and/or function of other immune or stromal cell types that reside in the TME.

Metabolic reprogramming of macrophages has been appreciated as an important factor for effective immune responses, including those that combat tumor growth, and our findings indicate that IFN/STAT1-induced NAMPT is a key component of this response. A majority of prior work on NAMPT and the NAD salvage pathway in macrophage inflammation have focused on the phenomenon of increased aerobic glycolysis under the influence of only LPS-based signaling^55–57^, while a few studies have started to address the role of IFNγ in pro-glycolytic transitions^9^. Although the de novo pathway of NAD biosynthesis has recently been shown to respond to inflammation and is a major contributor to NAD production in some contexts, NAMPT-mediated salvage synthesis of NAD is a critical pathway maintaining the NAD resource in many important cell types^58,59^. Our current study examined the impact of IFNγ driven classical M1 macrophage activation on the upregulation of glycolysis and inflammatory gene expression. It defines an important role for the direct IFNγ/STAT-dependent induction of the salvage NAD synthesis pathway during this process. A recent study is in agreement with our findings in terms of induced Nampt supporting glycolysis, where NAD is needed at the GAPDH step in glycolysis under LPS-only induction in macrophages. However, our study clearly expands on this concept and is unique in that it examines the metabolic roles of *Nampt* induction directly by IFNγ-dependent Stat1 recruitment to NRE1 and the effects of this phenomenon in the context of the immune cells in the TME, including melanoma patients. However, we also do not rule out possible roles for NAMPT-dependent NAD in the regulation of other molecular mechanisms relevant to macrophage biology, such as those involving Sirtuins^56,60^, PARPs^61–63^, and CD38^47,64^, and these will be explored in greater detail moving forward.

Our work also indicates that optimal induction of several macrophage inflammatory factors involves NAMPT regulation of glycolysis during the IFN response. One of these is the chemokine receptor CCR1, which functions within macrophages and other innate immune cells to promote homing to sites of inflammation, including invasion into tumors, and is vital to both immune cell homeostasis and the resolution of inflammation^65^. CCR1 in the TME may have both anti- and pro-tumorigenic activities, thus its characterization here as an inducible-*Nampt*-dependent factor and player in TAM recruitment is important. NRE1-mediated induction of *Nampt* is also important for other important inflammatory responses in macrophages, such as IL-6, IL-12p40, and *miR155* expression, in addition to NO production, all of which play critical roles in modulating antitumor immunity^4,35,66–69^.

NAMPT has previously been shown to mediate both cell intrinsic and extrinsic effects on cellular behavior. We observed decreased NAMPT enzyme and target gene expression in NRE1-deficient, IFN-activated macrophages, indicating that STAT1/NRE1-dependent induction of *Nampt* supports the inflammatory response. This, and the ability of FK866 to phenocopy these gene expression patterns yet be rescued by NMN, also point to a role for intracellular as opposed to extracellular NAMPT (eNAMPT)^70,71^ in our system. However, we cannot rule out any contribution by eNAMPT within the scope of the current study. Additionally, there may be many other factors at play in the exogenous addition of downstream metabolites of an inhibited enzyme, such as availability at needed locations and concentrations on the subcellular scale. Inhibited NAMPT may be incapable of carrying out other unknown functions unrelated to NMN production, or buildup of NMN precursors such as NAM in the presence of inhibited NAMPT may lead to allosteric action on gene regulatory processes. These possibilities will need to be evaluated in future work.

NAMPT has been widely explored as a target for tumor chemotherapy^18,19,22,46,47,58,72,73^, and the inhibitor FK866 and other NAMPT-targeting drugs have been subjects of clinical trials, with the rationale of inhibiting NAD-dependent Warburg aerobic glycolysis of tumor cells^19^. However, gross interference with an enzymatic activity essential for normal cell viability is a significant drawback for any drug. Moreover, our experiments indicate that NAMPT inhibitor treatment, even at doses without broad side effects, may be counter-therapeutic because of the critical roles for NAMPT induction in supporting pro-inflammatory immune responses against malignant disease, either endogenous or especially in relation to cancer immunotherapies. Specific enhancement of NAMPT induction in immune populations during interventional treatment may be one way to improve antitumor immune responses despite immunosuppressive stimuli in the TME. Assigning causality to the positive correlation between an immune-rich TME, inflammatory macrophage metagene signatures, high IFNγ-induced immune *NAMPT* expression, and tumor control will require further investigation. Additionally, strong correlation of IFNγ-induced signatures with *NAMPT* expression in multiple tumors suggests that this pathway of potential tumor control occurs more generally.

Ongoing research will also focus on understanding the roles of this pathway in other immune cell types, and in contexts where these cells promote autoimmune disease, pathogen clearance, or tumor surveillance. Along these lines, NAD metabolism was recently shown to play a role in mediating inflammation in a mouse model of inflammatory bowel disease, and the authors also demonstrated a role for enzymatic NAMPT function in promoting inflammatory macrophage differentiation and the expression of inflammatory genes in mice treated with FK866^74^. NAMPT has also been implicated in mediation of other inflammatory conditions including neuroinflammation, atherosclerosis, and arthritis^75–77^, and future work will take a closer look at the relevance of Stat1-induced NAMPT in these settings.

Our observation that dysregulation of *Nampt* narrowly in macrophages has a stronger defect in tumor control than the phenotype of a broader hematopoietic deletion that includes macrophages as well as all other leukocyte lineages suggests additional complexity in the role of *Nampt* induction in immune cells. It may be the case that inducible *Nampt* function in negative regulatory cell types or otherwise during hematopoiesis is also important during the evolving antitumor response^23,38,39^. Myeloid cells in the TME may be characterized by a mixed continuum of tumoricidal or pro-tumorigenic signatures, leading to complex heterogeneity in their behavior and interactions, and suggesting that more narrowly focused interrogation of the functions of NRE1 and similar regulators will be informative in future work.

Taken together, a model is emerging whereby IFN/STAT1/NRE1-upregulated *Nampt* functions to sustain NAD levels needed for optimal antitumor myeloid cell responses. As IFNs are key drivers of inflammation in a variety of contexts, our work suggests that a key function of IFNs is upregulation of NAD production via NAMPT, which is needed to drive both aerobic glycolysis and pathways required to unleash an inflammatory program contributing to antitumor mechanisms. As we continue to understand the role of inducible *Nampt* and NAD during macrophage and other immune cell responses, novel therapeutic regimens will undoubtedly emerge.

## Methods

### Mice

All mice are on the C57BL6 genetic background. IFNγR knockout mice and their wild-type controls were obtained from The Jackson Laboratory (Bar Harbor, ME), cataloged as B6.129S7-Ifngr1^tm1Agt^/J, Stock No: 003288. Conditional floxed NRE1 (NRE1 fl/fl) mice were custom generated by Biocytogen (Worcester, MA) using homologous recombination in C57BL6 ES cells. The final recovered mouse line carries the left NRE1 boundary (5′) loxP L containing cassette inserted into the genome at Chr12 between nt 32,821,032 and 32,821,033, and the right NRE1 boundary (3′) loxP R containing cassette at Chr12 between nt 32,827,113 and 32,827,114. A sequence file of the floxed locus is available on request. Male NRE1 fl/fl mice were then bred to Vav-iCre bearing females to generate hematopoietic specific NRE1 deleted mice hemizygous for Vav-iCre, and NRE1 floxed only or hemizygous Vav-iCre only controls. NRE1 deletion results in removal of 6080 bp of *Nampt* intron 1 in the target cell genome. An identical strategy to generate macrophage lineage-specific ablation of NRE1 instead used females carrying the LysM-Cre driver heterozygous with the WT *Lyz2* (LysM) allele. Mice were sex and age matched in each individual experiment, in vivo studies used females, and ages ranged from 6 to 10 weeks old.

Mouse vivarium housing conditions are maintained with a 12-h light/dark cycle per day, temperature of 22 °C, and 20% relative humidity. All housing, husbandry, and experimental procedures using mice were performed with the approval of the Institutional Animal Care and Use Committee and the Comparative Medicine Center of the University of Utah.

### Cell culture

Macrophage/myeloid cell lines including Raw 264.7, MV-4-11, Molm-14, and THP1 cells were cultured in D10 (DMEM media supplemented with 10% FBS, 1% Pen/Strep, and 1% L-glutamine). For generation of BMMs, bone marrow was isolated from the femurs and tibias of B6 WT or NRE1-KO mice and following RBC lysis were plated in cell culture plates at a concentration of 1–2 × 10^5^ cells per cm^2^ of growth area in D10 supplemented with 20 ng/ml M-CSF (Biolegend Cat #576404 or eBioscience Cat #14-8983-80) as described^78^. Cells were cultured 3 days before supplementing with additional media containing 20 ng/ml M-CSF, equivalent to half the amount of media added at day 0. Cells were utilized for experiments 6–7 days after differentiation with M-CSF. For the macrophage skewing RNA sequencing experiment, M1 skewed cells were stimulated with IFNγ (50 ng/ml, eBioscience Cat #14-8311-63) and LPS (10 ng/ml) and M2 skewed cells were stimulated with IL-4 (100 ng/ml, eBioscience Cat #14-8041-80) for 6 h. In experiments with IFNγ stimulation only, cells were stimulated with 50 ng/ml IFNγ, unless otherwise indicated.

Stimulation treatments were with LPS (10 or 100 ng/ml), IFNγ (50 ng/ml, eBioscience), IFNβ (10 or 50 ng/ml, Biolegend), poly I:C (10 ng/ml), Flagellin (1 ng/ml), CpG class A (1 µM), or pam3csk4 (1 ng/ml), unless otherwise noted, and were all obtained from Invivogen. Experiments utilizing RAW 264.7 cells were done similarly, except where as indicated, they were pretreated in triplicate with Ruxolitinib (Invivogen) at indicated concentrations (1000, 100, 10 nM, or DMSO vehicle control) for 1 h prior to addition of IFNγ. RAW 264.7 or BMM cells were stimulated with varied concentrations of the NAMPT inhibitor FK866 (10 nM–100 µM) or DMSO vehicle, and ±IFNγ (50 ng/ml) for 6–24 h for the analysis of RNA expression and protein expression via flow cytometry. Cells were not pretreated with the inhibitor prior to stimulating with IFNγ. Where indicated, 100 µM NMN was added to cell culture at the beginning of each experiment to rescue the effects of FK866 on the depletion of NAD. For protein, enzyme or metabolic activity, metabolite, and secretion assays, 10 µM FK866, 100 µM NMN, 10 ng/ml LPS, and/or 10 ng/ml INFγ were added simultaneously 24 h prior to analysis unless otherwise indicated. RNA measurements were done similarly except the times of treatments were 6 h.

### RNA isolation and RT-qPCR

For RNA analysis of TAMs, WT, and IFNγR-deficient mice were injected subcutaneously in the hind flank with 1 × 10^6^ B16f10 melanoma cells. After 12 days, tumors were resected and processed into a single-cell suspension. Cells from tumors in three to four mice were pooled and sort isolated by flow cytometry based on the expression of CD45, CD11b, and F4/80. Sorted TAMs were then placed in Qiazol and RNA was isolated with a miRneasy kit from Qiagen. Primary cell and cell line cultures were lysed with Qiazol reagent prior to isolation of total RNA with a miRNeasy RNA isolation kit (Qiagen).

In total, 100–400 ng of RNA was utilized in a cDNA synthesis reaction with qScript kit reagents (Quanta Biosciences) and was diluted to a final volume of 100–200 µl. Quantitative PCR (qPCR) was performed in a Lightcycler LC480 (Roche) or a QuantStudio 6 (Thermo) utilizing Promega GoTaq qPCR master mix reagents or the equivalent, and qPCR primers listed in Supplementary Table 1. RT-qPCR data presented are representative of at least two independent experiments with at least three biological replicates per condition, and two to three technical replicate PCR reactions per sample. Gene symbols for target genes analyzed are listed in Supplementary Table 2.

### CRISPR/Cas9-mediated gene deletion

CRISPR/Cas9 lentivector infections were performed by utilizing Trans-IT 293 (Mirus) to transfect 293T cells with the packaging plasmids pVSVg and psPAX2 along with the CRISPR/Cas9 lentiCRISPRv2 construct (Addgene plasmid #52961) containing one specific short guide RNA (sgRNA) sequence and either a GFP or Puromycin selection marker, as described^69,79^. To generate NRE1-KO cells, RAW 264.7 cells were transduced with two separately marked sgRNA expressing lentiviral constructs and selected by both puromycin resistance and GFP expression. These are labeled NRE1 CR. Clonal cell populations were obtained by plating single cells on a 96-well plate and the expression of NRE1 was quantified via RT-qPCR to verify knockout of NRE1. Deletion of the SBS within NRE1 in RAW 264.7 cells was performed similarly except two sgRNAs targeting sequences immediately flanking on either side of the SBS region (Stat1 BS CR) were expressed from one construct via a dual promoter system. This vector was designed at the University of Utah Mutation Generation and Detection Core, consisting of the U6 promoter for one gRNA and the H1 promoter for the second, all in a lentiCRISPRv2 derivative. *Stat1* knockout RAW 264.7 cells were generated by targeting a single sgRNA to either exon 3 (*Stat1*-CR1) or exon 4 (*Stat1*-CR2) of the *Stat1* gene. Controls for each of these cell lines were generated in a similar manner as described above with a CRISPR/Cas9 lentiCRISPRv2 empty vector plasmid that contained no sgRNA sequence with the exception of the controls for the SBS deletion within NRE1 which had a non-targeting sgRNA sequence. sgRNA sequences are described in Supplementary Table 1.

### Genotyping of NRE1-KO cell lines

NRE1 CRISPR/Cas9 knockouts in Raw cells were genotyped by PCR using NRE1 5′ CRISPR Site F and NRE1 3′ CRISPR Site R primers (Supplementary Table 1) to amplify a 300 bp product from genomic DNA if the NRE1 region had been deleted. In the case where cells had a heterozygous genotype, PCR of the region surrounding the CRISPR-Cas9 binding sites was performed using NRE1 5′ CRISPR Site F and R, and NRE1 3′ CRISPR Site F and R primers (Supplementary Table 1). The PCR products or their TOPO vector clones were analyzed by Sanger sequencing to identify mutations found in these regions.

### Western blots

Cell pellets were lysed in RIPA buffer containing 150 mM Tris (pH7.4), 150 mM NaCl, 1% NP-40, 1 mM EDTA, and 1% SDS. Purified protein was separated via SDS-PAGE and transferred to a 0.45 µM nitrocellulose membrane. Antibody staining of NAMPT (Bethyl Laboratories), STAT1 (Santa Cruz sc-346), and ACTB (Santa Cruz sc-47778) was performed. HRP conjugated secondary antibody was detected with Amersham ECL reagent (GE) with Biorad GelDoc XR+ and ImageLab 6.1 software. Uncropped images of blots are included in the Source Data Supplementary file.

### ChIP

ChIP was performed as previously described^80^ with modifications. 5 × 10^7^ RAW 264.7 cells per sample in 15 cm tissue culture plates treated with 50 ng/ml IFNγ or medium were crosslinked for 15 min at 37° in PBS with 5 mM EDTA and 1% paraformaldehyde, quenched with glycine, and lysed at 0° in ChIP Lysis Buffer (FA buffer containing 0.1% NP-40, 0.1% SDS, 2 mM MgCl_2_, 1 mM DTT, and protease inhibitors, using zircon beads with a Mini-Bead-Beater 96 (Biospec Products)). Washed lysate pellets were resuspended in fresh ChIP Lysis Buffer and sonicated using a Bioruptor XL bath sonicator (Diagenode). Soluble chromatin containing DNA sheared to 200 bp average length was immunoprecipitated with anti-STAT1 (sc-529, Santa Cruz) bound to Protein-G magnetic beads^81^. Bound chromatin was de-crosslinked in 1% SDS, 50 mM tris pH 8.5, 10 mM EDTA, and purified with Qiaquick PCR Columns (Qiagen). Aliquots of each sample’s total input chromatin prior to immunoprecipitation were de-crosslinked in parallel.

Chromatin occupancy was then determined by quantification of bound chromatin relative to the total input, performed by preparing a dilution series of the input total chromatin reactions analyzed by qPCR as above in parallel to the ChIP and input samples, to generate a standard curve. Each sample’s concentration was determined from the reactions’ Cq using the manufacturer software. Levels of occupancy were compared between samples by first calculating a ChIP signal by dividing the relative concentration of the indicated targets in the ChIP reaction by that of the input chromatin to obtain a fraction or % IP value, then normalizing each ChIP signal to that for a constitutively transcribed unbound region^82^ within the RpL32 reference gene to obtain fold enrichment values. Error bars represent SEM of fold enrichment from the average of three separate cell cultures. ChIP Primer sequences are listed in Supplementary Table 1.

### Northern blots

Total cellular RNA was electrophoresed in denaturing agarose gels, then transferred to a nylon membrane by passive transfer overnight with 20X SSC, UV crosslinked, and prehybridized at 42 °C for 1 h using UltraHyb (Invitrogen). Random-primed DNA probes or antisense RNA probes were radiolabeled with ^32^P according to manufacturer’s instructions (Thermo Fisher) and hybridized overnight before washing with SSC/SDS. Bound probes were visualized using a Phosphorimager (GE Healthcare) and Imagequant. Uncropped images of blots are included in the Source Data Supplementary file.

### Conservation of binding motifs

For the analysis of species conserved STAT1 consensus binding elements of the *Nampt* NRE1 in Fig. 3d, sequences from corresponding regions of intron 1 from the listed mammals were downloaded from the most current assemblies using the UCSC genome browser (http://genome.ucsc.edu). Segments of these conserved regions were aligned using ClustalX 2.1, with default parameters except Gap Opening and Extension penalties set to 5 and 3.33, respectively. Edited msf output files were then formatted with Boxshade (http://www.ch.embnet.org/software/BOX_form.html). Stat binding sequence motifs (red box) were identified in the *Nampt* locus using the JASPAR transcription factor binding motif database (http://jaspar.genereg.net)^26^. Base numbers are from Mouse *Nampt* TSS =+1.

### Luciferase assay

A 280 bp fragment of the WT *Nampt* Intron 1 NRE1 containing bp 696–975 relative to +1 of the *Nampt* mRNA and including STAT1 binding motifs was assembled into the *Oplophorus* luciferase (NanoLuc) vector pNL3.2 (Promega, GenBank JQ513370.1). A mutant version of this region was synthesized with the same length and G + C content but with the STAT1 motifs abolished based on Shuffleseq (EMBOSS: www.bioinformatics.nl/cgi-bin/emboss/shuffleseq)^83^, and was confirmed to have no STAT1 binding sequences using JASPAR (http://jaspar.genereg.net/)^26^. In total, 250 ng of WT or mutant plasmid, pNL3.2 alone, or a TK-NanoLuc positive control plasmid were each transduced along with 250 ng pGL4.54 *Photinus* luciferase reference plasmid into 5 × 10^5^ RAW 264.7 macrophage cells using Lipofectamine LTX (Thermo Fisher) according to manufacturer’s directions for 8 h allowed to recover for 12 h in fresh medium, then treated with IFNγ or medium alone for 6 h. Lysates prepared from these cells were assayed for luciferase activity using the Nano-Glo Dual-Luciferase Reporter Assay System (Promega) in a Synergy H1M plate reader (Biotek).

### ECAR and OCR assay

An Agilent Seahorse XF-96 Analyzer was used to perform the ECAR (extracellular acidification rate) or OCR assays under conditions of glycolytic (GST) or MST in the Metabolic Phenotyping Core Facility at the University of Utah. BMMs obtained as above were plated on 96-well Seahorse XF Cell Culture Microplates the day prior to performing the assay. Seahorse XF Base Medium was supplemented with 1 mM glutamine, adjusted to a pH of 7.4 with 0.1 N NaOH, and filtered through a 0.22 µm filter for use during the assay. For ECAR/GST, 10 mM Glucose, 1 μM oligomycin, and 50 mM 2-DG were, respectively, added to ports A, B, and C of the analyzer, then each solution was added at the indicated time points. For OCR/MST, 1.5 μM oligomycin A, 1.5 μM FCCP, and 2.5 μM antimycin A + 1.25 μM rotenone were added as indicated.

### Mass spectrometry

Metabolomics analysis was performed by the Metabolomics Core Facility at the University of Utah. Cells from the indicated treatment conditions were detached in ice cold PBS with 5 mM EDTA, collected, and centrifuged at 21,000 × *g* for 1 min, supernatant aspirated, and cell pellets were snap-frozen with liquid nitrogen. Cold 90% methanol (MeOH) solution containing the internal standard d4-succinic acid (Sigma 293075) to give a final concentration of 80% MeOH was added to each cell pellet. Samples were then quickly vortexed, sonicated for 5 min., incubated at −20 °C for 1 h, then centrifuged at 20,000 × *g* for 10 min at 4 °C. Appropriate quality control samples were made in parallel. All GC-MS analysis was performed with an Agilent 7200 GC-QTOF and an Agilent 7693A automatic liquid sampler. Dried samples were suspended in 40 μl of 40 mg ml^−1^ O-methoxylamine hydrochloride (MOX) in pyridine and incubated for 1 h at 30 °C. Metabolites were identified and their peak area was recorded using MassHunter Quant (Agilent). Metabolite identity was established using a combination of an in-house metabolite library developed using pure purchased standards, the commercially available NIST library, and the Fiehn library.

For NAD+ measurement by liquid chromatography–mass spectrometry (LC-MS), cell pellets were extracted using a chilled solution of 100 µl of 90:10 methanol:water containing five nanograms of d9-carnitine (Cambridge Isotope Laboratories) as an internal standard. Each sample was vortexed for 30 s and centrifuged for 10 min at 20,000 RCF at 4°. The supernatant was removed to a fresh tube and dried overnight en vacuo. Immediately prior to LC-MS, the samples were reconstituted in water containing 10 mM of ammonium carbonate. Mass spectral analysis was performed using an Agilent 6550 UPLC-QTOF-MS system (Santa Clara, CA). A Hiliocon iHILIC column (Umea, Sweden) was used for fractionation using a linear gradient of 10:90 ACN:10 mM ammonium acetate buffer to 20:80 ACN:10 mM ammonium acetate buffer over 20 min at a flow rate of 0.2 ml/min. MS was performed in the positive mode with an AJS ESI source. Quantitative data analysis was conducted using Agilent MassHunter Quant software for NAD+.

### RNA sequencing

Total RNA isolated via miRNeasy Qiagen RNA isolation kit was submitted to the University of Utah genomics core facility, a cDNA library was prepared and Illumina HiSeq 2500 sequencing was performed as in^67^, with modifications. Briefly, RNA was quantified with a Qubit RNA HS Assay Kit (Fisher Scientific #Q32855). RNA quality was evaluated with an Agilent Technologies RNA ScreenTape Assay (5067-5579 and 5067-5580). After rRNA depletion reactions on 100–500 ng total RNA, stranded libraries were prepared using the Illumina TruSeq Stranded Total RNA Kit with Ribo-Zero Gold (RS-122-2301 and RS-122-2302). Purified libraries were qualified on an Agilent Technologies 2200 TapeStation using a D1000 ScreenTape assay (5067-5582 and 5067-5583). Individual libraries were normalized to 10 nM and equal volumes were pooled in preparation for Illumina sequence analysis. Libraries (25 pM) were chemically denatured and applied to an Illumina HiSeq v4 single read flow cell, amplified and annealed to sequencing primers, and 50 cycle single read runs were performed using an Illumina HiSeq 2500 instrument (HCSv2.2.38 and RTA v1.18.61), and corresponding reagent kits (GD-401-4001, FC-401-4002). Resulting data were aligned to the mouse mm10 reference genome (mm10, M_musculus_Dec_2011, GRCm38).

### scRNAseq and syngeneic tumor experiments

Mice were injected subcutaneously in the rear flank with 5 × 10^5^ B16F10-ova Melanoma or MC-38-ova COAD cells on day 0^35^. Tumor size measurements were taken on the indicated days, and tumors were weighed upon sacrifice at day 14. Dimensions (mm) were measured using calipers, and size was estimated using the formula: (*l* × *w*^2^)/2, reported as Tumor Size. For further analysis, tumors were mechanically disrupted using the frosted ends of microscope slides and treated with Accumax (Innovative Cell Technologies, CA) for 30 min as reported previously^35^. Homogenized tumors were pooled per genotype and samples were stained with fluorophore-conjugated anti-CD45 antibodies for 15 min in PBS containing 0.5% BSA and 2 mM EDTA followed by flow cytometric sorting. DAPI-negative live CD45+ cells were sorted and used for scRNAseq via 10X platform (High-throughput Genomics Core, University of Utah). Analysis of scRNAseq data was done using the Seurat R package^36,84^. Briefly, low quality cells (<200 total features and >5% mitochondrial gene frequency) were discarded. WT and NRE1-KO data were integrated using variable genes and 20 metagene dimensions (determined by examining flattening of the standard deviation elbow-plot), followed by cluster identification. Clusters were named as done previously using an in-house developed algorithm that scores unknown cluster gene signatures against the known immune cells in the ImmGen database. Closely related clusters were distinguished and named accordingly by the help of pairwise differential expression analyses.

The computational approach we used for annotating unknown cell clusters (Cluster Identity Predictor (CIPR)) was previously described^34^. In short, the CIPR algorithm compares the genome-wide expression signatures from unknown single-cell clusters to that of known reference cell types published by the ImmGen consortium. The reference dataset contains microarray data from sorted mouse immune cell populations. By examining several genes simultaneously, CIPR calculates a similarity score for each single-cell cluster and known reference pairs to aid quickly and accurately annotate unknown clusters through CIPR-Shiny (v0.1.0), a user-friendly graphical interface available on the web (https://aekiz.shinyapps.io/CIPR/)^34^. The CIPR algorithms implement refinements to some cell cluster assignments relative to older established pipelines (such as SingleR R package), and with less computing resource requirements.

GO analysis was performed on differentially expressed gene sets in WT and NRE1-KO cell clusters in scRNAseq data. To this end, the FindMarkers() Seurat function was used to calculate upregulated genes in WT or NRE1-KO single-cell clusters through Wilcoxon Rank Sum test (default). The significance cutoff for subsequent GO analysis was set at the unadjusted *p* value of <0.05. These gene sets were then analyzed using the clusterProfiler R package against Biological Process GO terms. GO term enrichment results are presented in tabular format for each cluster (Supplementary Data 2 and Supplementary Data 3).

### Flow cytometry

Flow cytometric analyses were performed on cultured cell lines and single-cell suspensions isolated from tumors, and carried out as described in^85^ with modifications. Cultured cells were treated with various reagents as described and were collected off the plate via 5 mM EDTA in PBS in a non-enzymatic manner. Briefly, after mechanical disruption and dissociation of tumor tissue between the frosted ends of microscope slides, tumor cells were placed on an orbital shaker in Accumax (Innovative Cell Technologies) and incubated for 30 min at room temperature, followed by red blood cell lysis in ammonium-chloride-potassium buffer (Biolegend), and filtration through a 0.45-micron filter. Prior to staining with fluorophore-conjugated antibodies (all from Biolegend or Thermo/eBioscience), Fc receptors were blocked using αCD16/32 antibody (1:200, Biolegend) to reduce nonspecific staining. Staining was performed on ice for 15–30 min in PBS supplemented with 2% FBS and 0.1% Sodium Azide. Cells were stained with a combination of the following fluorophore-conjugated antibodies in Hanks’ balanced salt solution supplemented with 10% BSA, pyruvate, EDTA, and HEPES: CD45 PE-cy7 (1:400), CD45 PB (1:400), Ly6c FITC (1:200) MHCII PB (1:200), Gr1 PE (1:2000), CD40 FITC (1:200), CD86 Percp-cy5.5 (1:200), F4/80 PE (1:200), F4/80 APC (1:200), CD11b Percp-cy5.5 (1:200), CD11b APC (1:200), all from Biolegend unless noted otherwise. For viability assays, Ghost Dye Red 780 (Tonbo) stain was applied. The cells were then washed, and flow cytometry was performed using a BD LSRFortessa II (BD Biosciences). Data were subsequently analyzed using FlowJo software (Tree Star). Gating strategies are provided as Supplementary figure panels corresponding to the relevant figures. Sorting of GFP+ cells was completed on a FACSAria III (BD Biosciences) in the flow cytometry core facility at the University of Utah.

### Analysis of TCGA data

SKCM bulk RNA-seq data were downloaded from TCGA database using TCGAbiolinks R package. Raw counts were normalized using the voom approach and *NAMPT* expression was categorized at median or top bottom 10% to define NAMPT-high and NAMPT-low patients. In rare cases where both primary and metastatic samples are available from the same patient, metastatic samples are used in the analyses. Immune rich vs. immune devoid (keratin signature) classifications were used according to published standardized criteria in TCGA^40^.

### Statistical analysis

T-Tests (two-tailed, unpaired) were performed when comparing differences between two experimental groups in in vitro experiments where noted in the figure legends. Two-way ANOVA was also used where noted. Wilcoxon test was used when calculating differential gene expression and comparing gene expression in scRNAseq data. GSEA profiles were evaluated using two-tailed *p* values corrected for multiple comparisons using Benjamini–Hochberg method. *p* values are reported in the figure panels for the difference between high and low *NAMPT* expression plots for either the keratin signature or immune signature-rich samples, and are from the log-rank (Mantel–Cox) test. Unless otherwise noted, the following symbols were utilized in all figures to denote significant *p* values: *p < 0.05, **p < 0.005, ***p < 0.0005, ****p < 0.00005, and ns for not significant. *p*_adj_ denotes false discovery rate in GSEA plots.

### Reporting summary

Further information on research design is available in the Nature Research Reporting Summary linked to this article.

## Supplementary information

Supplementary Information

Descriptions of Additional Supplementary Files

Supplementary Dataset 1

Supplementary Dataset 2

Supplementary Dataset 3

Reporting Summary

## Data Availability

RNA-seq and scRNAseq data have been deposited in Gene Expression Omnibus (GEO) under the following accession codes: GSE113836, GSE113837, GSE123822, and GSE135814. TCGA data are available at https://www.cancer.gov/about-nci/organization/ccg/research/structural-genomics/tcga. All other data supporting the findings of this study are available within the article and Supplementary information files. Source data are provided with this paper.

## Source data

Source Data
