# Supplementary material for: A Stat1 bound enhancer promotes Nampt expression and function within tumor associated macrophages
Source: Nat Commun. 2021 May 11;12:2620. doi: 10.1038/s41467-021-22923-5 (PMC8113251; doi:10.1038/s41467-021-22923-5)
Supplement: Supplementary file 1 — Supplementary Information [file 41467_2021_22923_MOESM1_ESM.pdf]

Supplementary Figure S1.

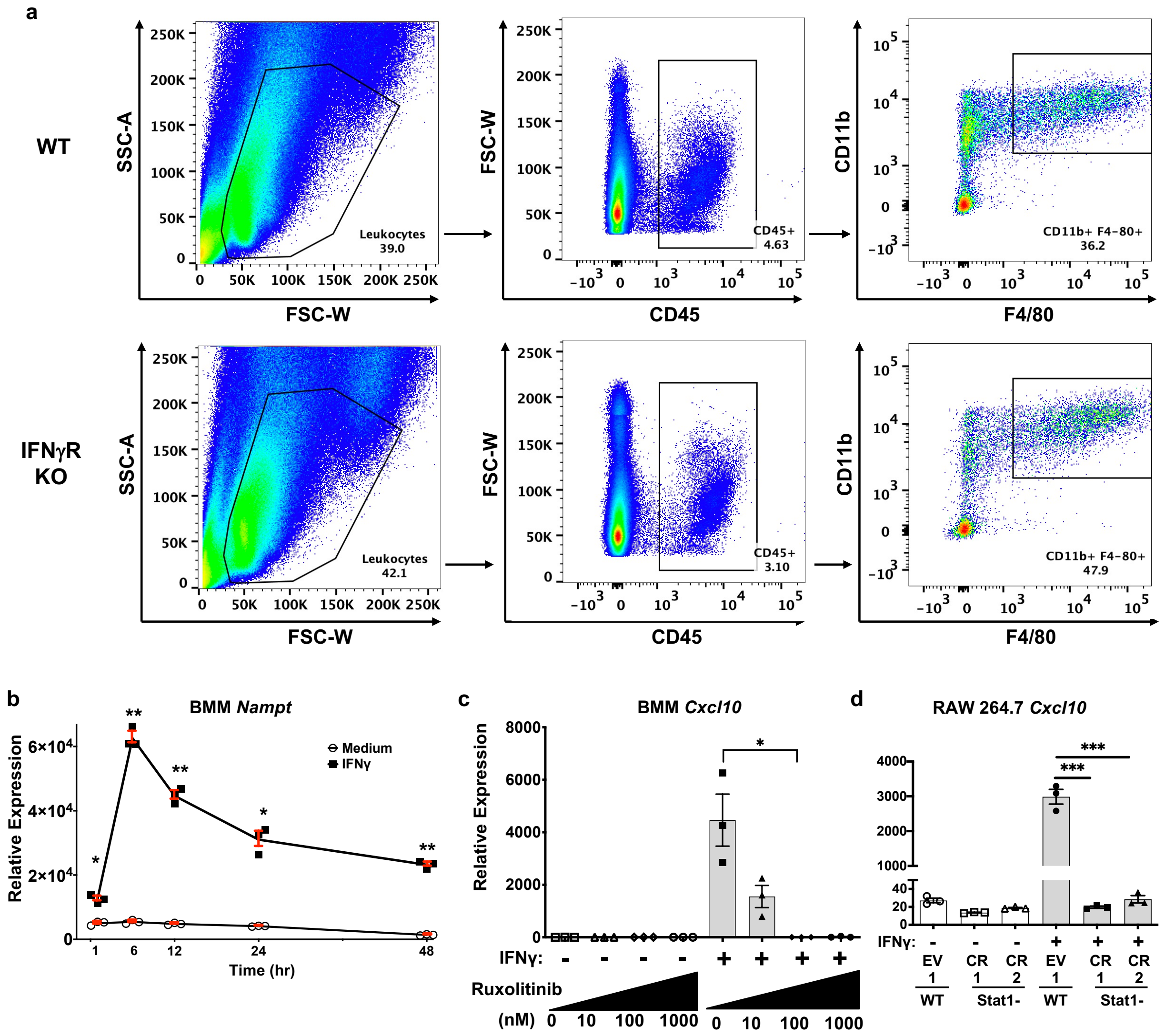

Supplementary Figure S1. Related to Figure 1. Macrophages upregulate *Nampt* in response to IFNγ through Jak/Stat1 signaling. (A) Flow cytometry staining and gating scheme for FACS isolation of TAMs from B16F10 tumors in WT or IFNγR knockout animals. Source data provided in Source Data file in Supplementary Information. **(B)** Induced expression timecourse of *Nampt* by qPCR performed on BMMs treated with IFNγ for the indicated times, shown as individual values with the mean for N=3 independent BMM samples, error bars (red) are SEM. *P* values were determined by a two-way ANOVA using Sidak's multiple comparison test: *p<.05, **p<.005, ***p<.0005, ****p<.00005. **(C)** Expression of *Cxcl10* in BMMs treated with Ruxolitinib at the indicated concentrations with and without IFNγ stimulation, as a control for inhibition of Nampt in Fig. 1G. **(D)** *Cxcl10* expression in RAW 264.7 cells in which STAT1 expression was deleted via CRISPR/Cas9 targeting of *Stat1*, as a control for loss of activation of *Nampt* in Fig. 1H. Results presented are the average of at least 3 biological replicates. Except for panel S1A, data are representative of at least 3 independent experiments. For **(B-D)**, data are the average of N = 3 biological replicates. P values were determined by a two-tailed t-test: *p<.05, **p<.005, ***p<.0005, ****p<.00005. Error bars represent SEM.

Supplementary Figure S2.

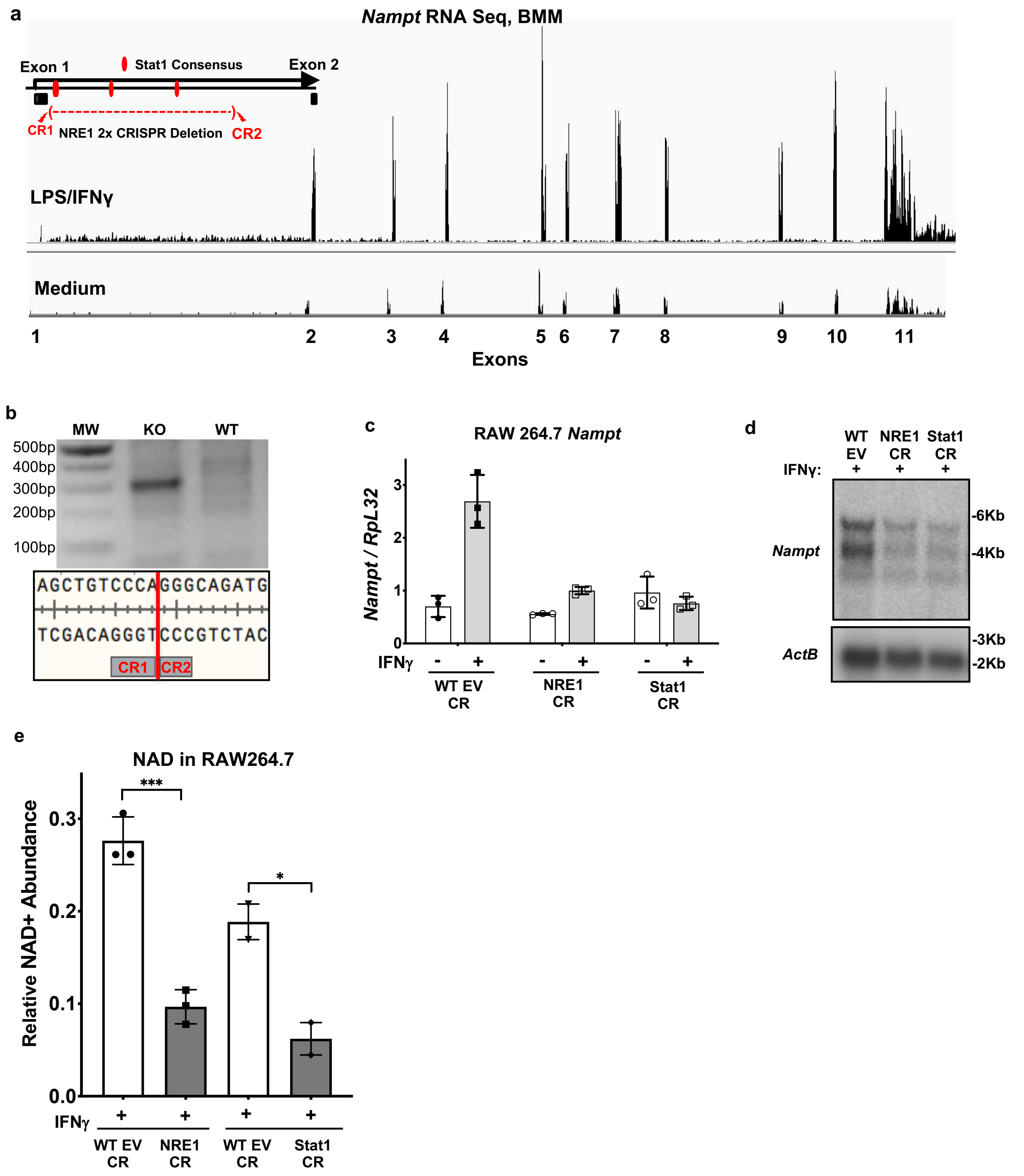

Supplementary Figure S2. Related to Figure 2. IFNγ-inducible expression of *Nampt* in macrophages through NRE1. (A) RNA-Seq tracks from the *Nampt* locus in BMMs treated with M1 conditions (LPS/IFNγ) or medium, showing elevated read levels in the first intron under activation. *Nampt* exons are designated by their number. **(B)** PCR of new junction sequence created by deleting NRE1 from genome in a RAW 264.7 cell clone with CRISPR-Cas9. A ~300

bp product was amplified from genomic DNA using primers (o899 and o900) flanking NRE1 boundaries when the ~5Kb NRE1 region was deleted. Sanger sequencing base calls identifying the location of the deletion endpoints. Gel shows results representative of 4 independent clones Size markers are in bp**.** **(C)** qPCR of *Nampt* expression in EV WT clones, NRE1 CR clones, and *Stat1* gene CR clones with and without IFNγ stimulation for 6 hours. **(D)** *Nampt* northern blot of IFNγ treated (6hr) WT EV, NRE1 CR, and *Stat1* CR Raw 264.7 cells. Size markers are in kB. For panels S2C-D, data are representative of at least 3 independent experiments. **(E)** LC-MS quantification of NAD+ in both NRE1 CRISPR knockout and Stat1 CRISPR knockout IFNγ treated RAW264.7 cells. EV: Non-Targeting CRISPR gRNA Control. For **(C, E)**, data are the average of N = 3 or 2 biological replicates. P values were determined by a two-tailed unpaired t-test: *p<.05, **p<.005, ***p<.0005, ****p<.00005. Error bars represent SEM or range. Uncropped images provided in Source Data file in Supplementary Information.

Supplementary Figure S3.

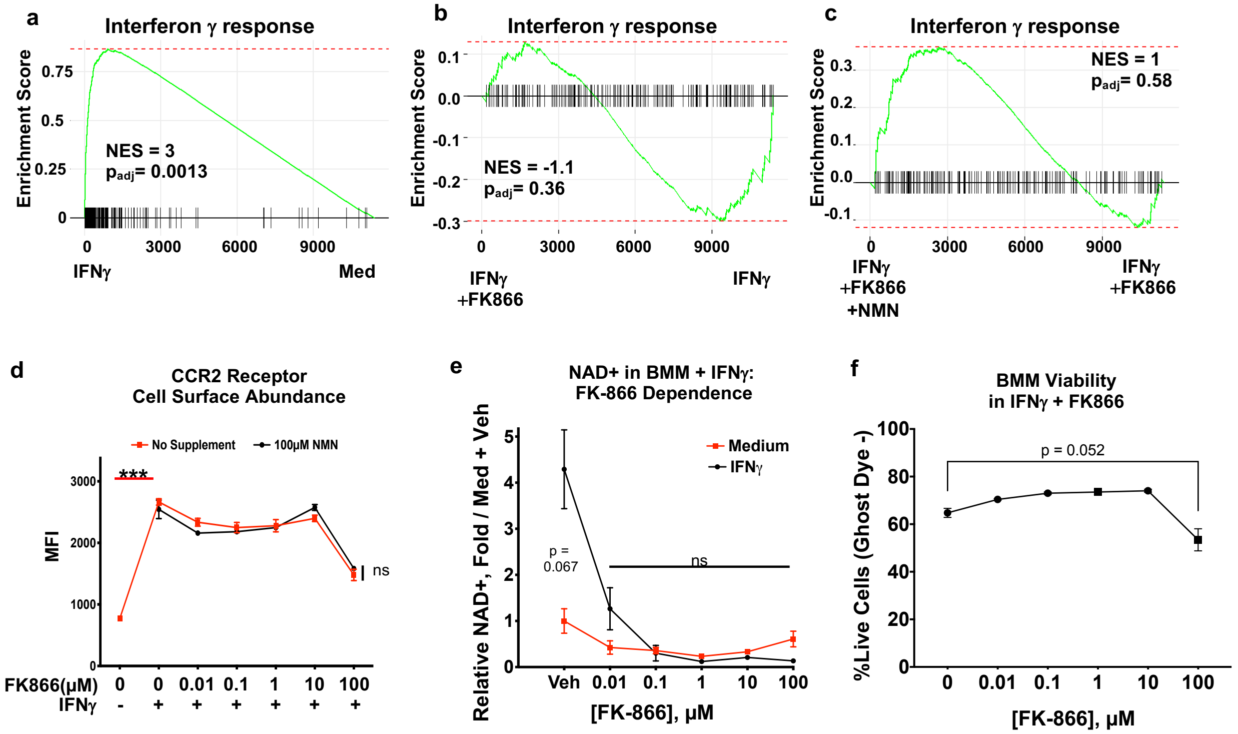

Supplementary Figure S3. Related to Figure 3. NAMPT is required for inflammatory gene expression programs during the IFNγ response. (A) The IFNγ response pathway GSEA gene set expression pattern in the presence of NAMPT inhibitor FK866, and also **(B)** FK866 but with restored NMN/NAD levels, in comparison with the **(C)** IFNγ response pathway gene set in samples subjected to IFNγ treatment alone. **(D)** CCR2 cell surface protein expression levels as measured by flow cytometry on BMMs treated with IFNγ and increasing amounts of FK866, as in Fig. 4D. **(E)** FK866 dose-dependent reduction in IFNγ induction of NAD+ levels in BMMs. **(F)** Ghost dye viability assays showing BMM viability in the presence or absence of IFNγ is not significantly affected by FK866 addition. For **(D-F)**, Data are the average of N = 3 biological replicates or 2 for NAD+ analysis. P values were determined by a two-tailed unpaired t-test: *p<.05, **p<.005, ***p<.0005, ****p<.00005. Error bars represent SEM or range.

Supplementary Figure S4.

a

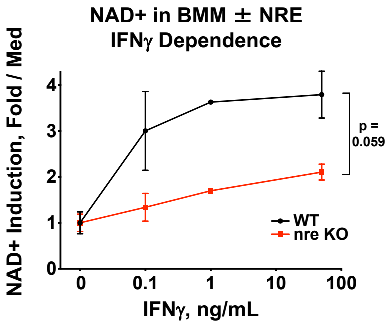

Supplementary Figure S4. Related to Figure 4. NRE1 is required for IFNγ inducible synthesis of NAD. (A) NAD synthesis in the presence of IFNγ is blunted in the absence of NRE1 in Vav-Cre NRE1 fl/fl (NRE1-KO) macrophages. NAD+ was measured by LC-MS in these cells treated with the indicated concentrations of IFNγ, and is reported as normalized to untreated cells of each genotype. Data are the average of N = 2 biological replicates. P values were determined by a two-tailed unpaired t-test: *p<.05, **p<.005, ***p<.0005, ****p<.00005. Error bars represent range.

Supplementary Figure S5.

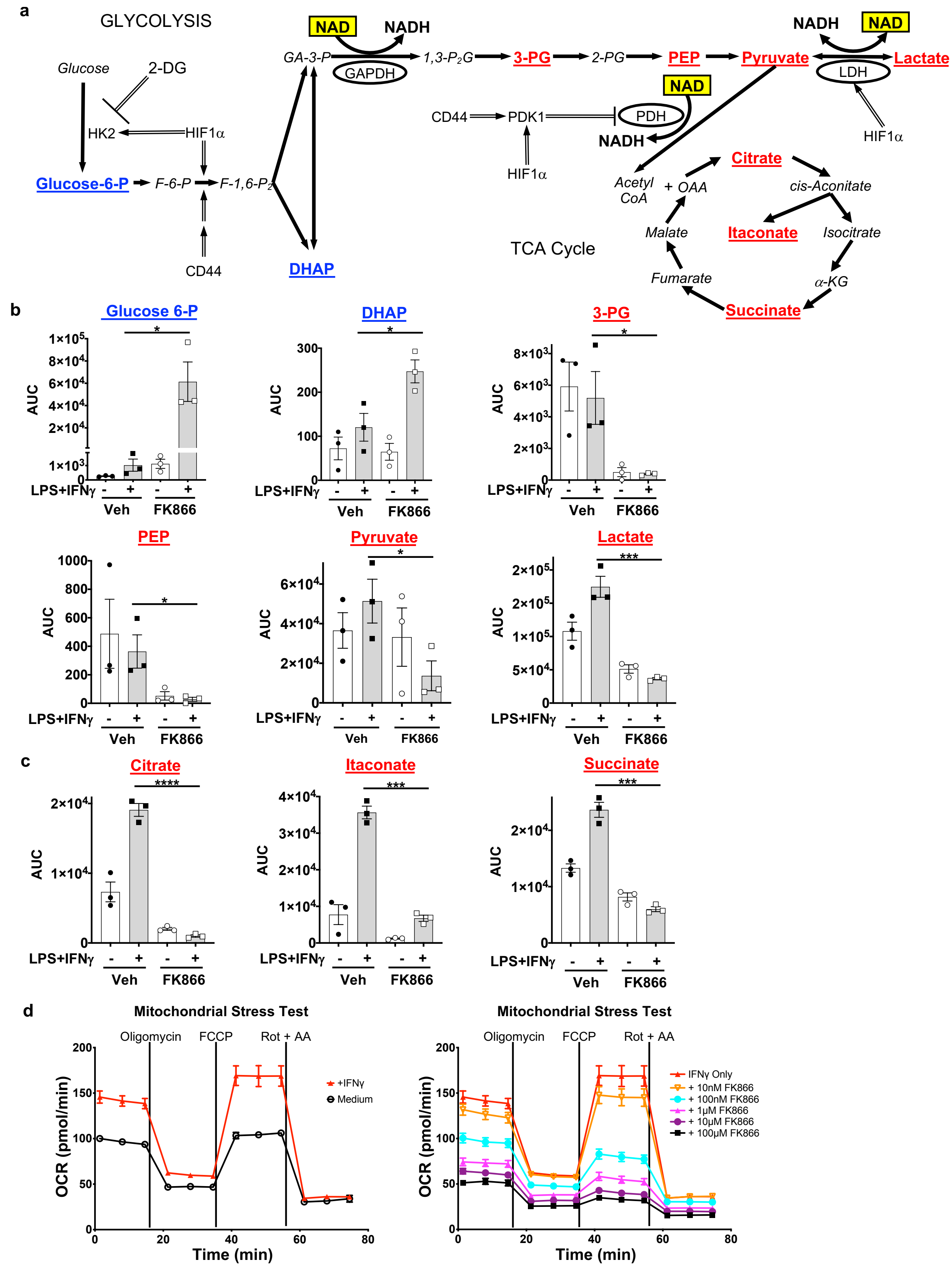

Supplementary Figure S5. Related to Figure 5. NAMPT enzymatic function is required for induced glycolysis at the GAPDH step in LPS/IFNγ stimulated BMMs. Individual data points for normalized ratio values plotted in Figure 5B. NAMPT function is required at the GAPDH catalyzed step in glycolysis in LPS/IFNγ stimulated BMMs. **(A)** Pathway schematic for glycolysis and the TCA cycle. Bold and underlining indicates the metabolites reported in (B-D) with names in blue for those that are upstream of the first NAD utilizing step, and in red for those that are downstream of NAD mediated steps, the points of NAD utilization with ovals indicating the enzymes with an NAD cofactor, and with positions of function for the regulatory protein factors which are analyzed in this study [86]. (B-C) Metabolomic analysis for intermediates detected by GC-MS in the **(B)** glycolytic and **(C)** TCA cycle pathways from BMMs treated with combinations of LPS/IFNγ and FK866, showing buildup of metabolites upstream of enzymes normally requiring NAD in the presence of stimulation and FK866 (red), and the reduction of induced intermediates downstream of those NAD requiring steps upon FK866 addition (blue). Results presented in **(B-C)** are the average of 3 biological replicates. AUC: Area Under Curve. **(D)** IFNγ stimulated increases in respiration under MST measured by OCR are also sensitive to Nampt inhibition by FK-866. Data are the average of N = 7 or 8 biological replicates. Error bars represent SEM. P values were determined by a two-tailed unpaired t-test: *p<.05, **p<.005, ***p<.0005, ****p<.00005.

Supplementary Figure S6.

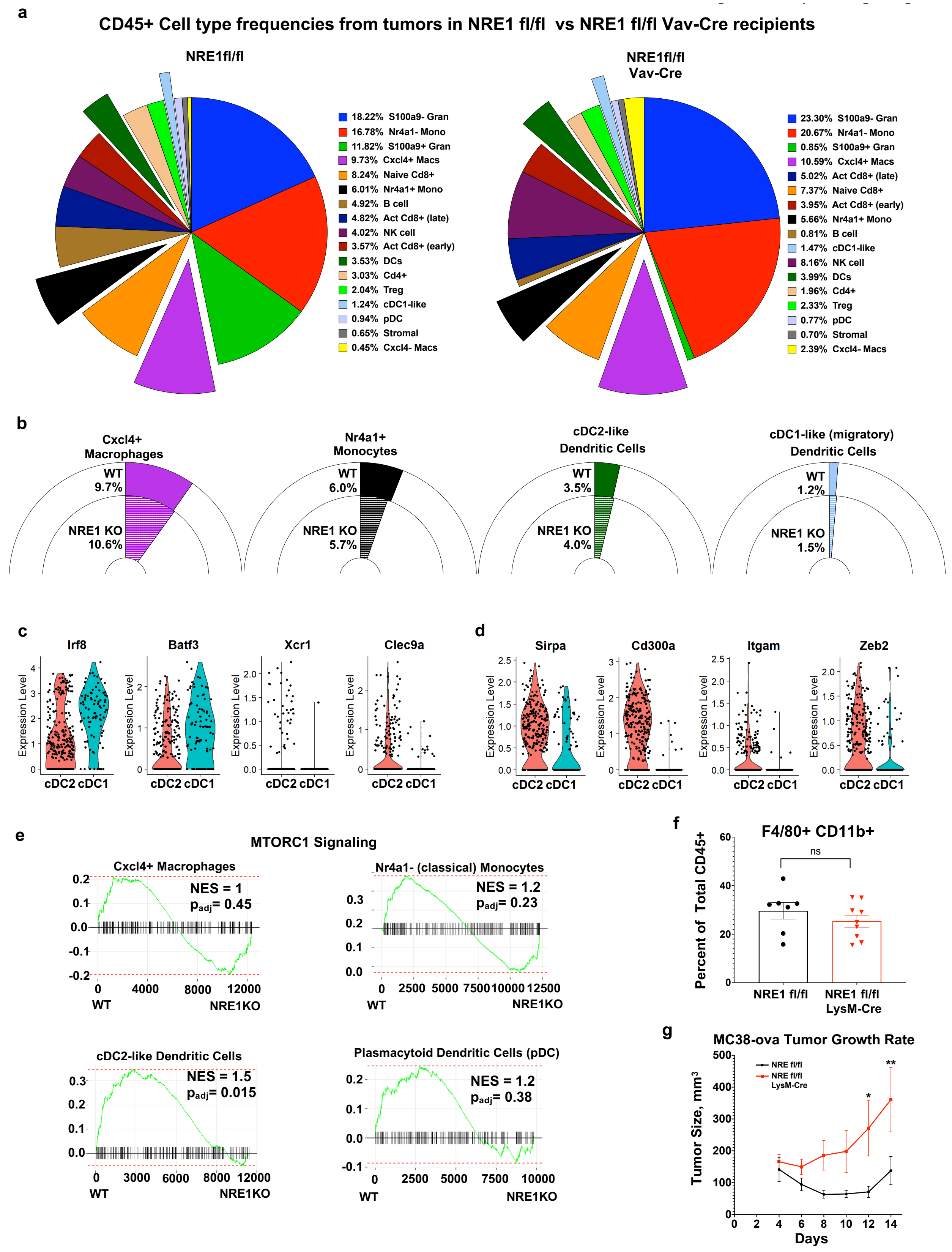

h

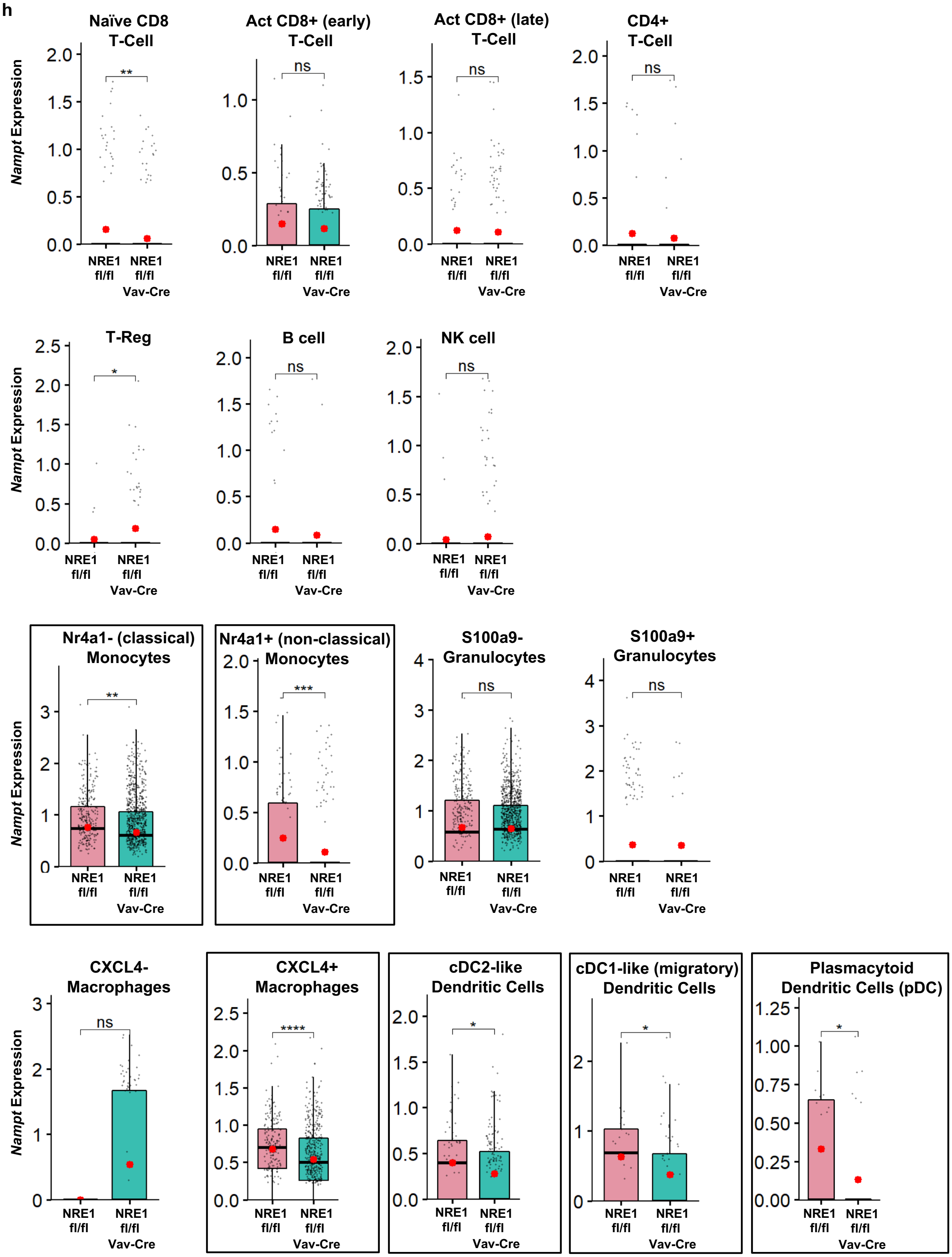

Naïve CD8 T-Cell
Act CD8+ (early) T-Cell
Act CD8+ (late) T-Cell
CD4+ T-Cell
T-Reg
B cell
NK cell
Nr4a1- (classical) Monocytes
Nr4a1+ (non-classical) Monocytes
S100a9- Granulocytes
S100a9+ Granulocytes
CXCL4- Macrophages
CXCL4+ Macrophages
cDC2-like Dendritic Cells
cDC1-like (migratory) Dendritic Cells
Plasmacytoid Dendritic Cells (pDC)
Nampt Expression
NRE1 fl/fl
NRE1 fl/fl Vav-Cre
ns
*
**
***
****

i

Flow Cytometry Gating Strategies for Figure 6b:
Relative abundance of CD45+ TILS in purification for scRNASeq was 36.4% (6.3% of Total) for WT, 22.4% (5.1% of Total) for NRE-ko.
Post-sorting purity was 96%.

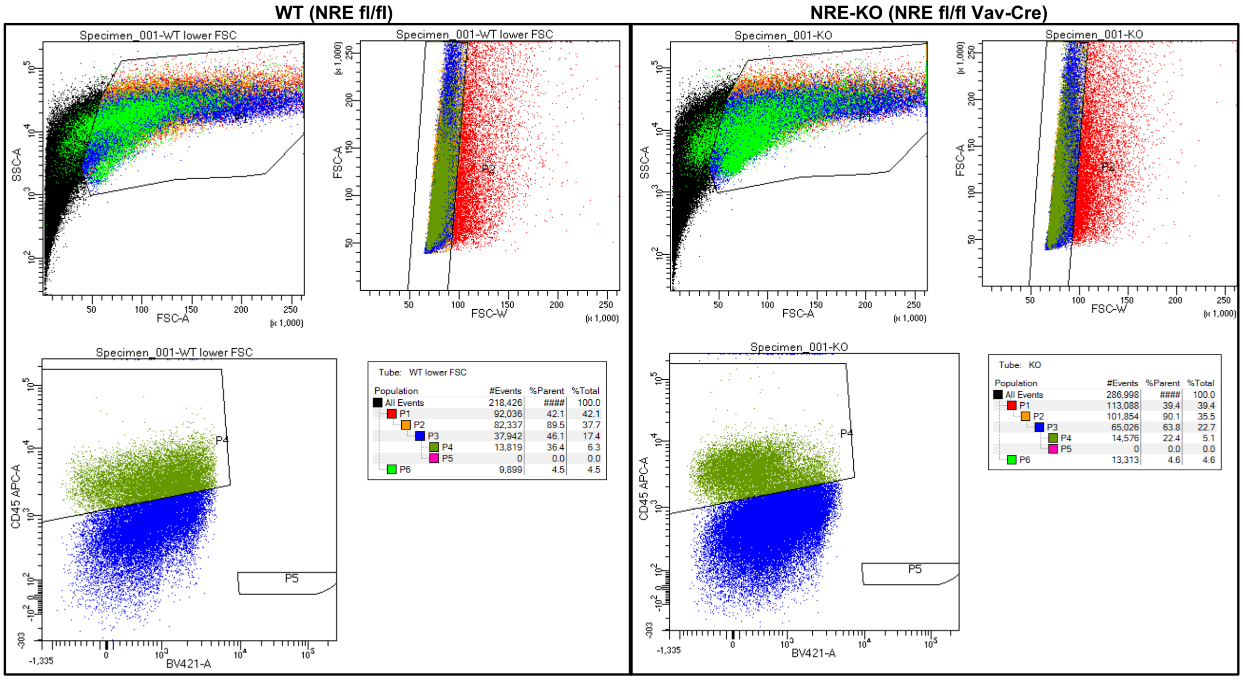

Tube: WT lower FSC

Population	#Events	%Parent	%Total
All Events	218,426	####	100.0
P1	92,036	42.1	42.1
P2	82,337	89.5	37.7
P3	37,942	46.1	17.4
P4	13,819	36.4	6.3
P5	0	0.0	0.0
P6	9,899	4.5	4.5

Tube: KO

Population	#Events	%Parent	%Total
All Events	286,998	####	100.0
P1	113,088	39.4	39.4
P2	101,854	90.1	35.5
P3	65,026	63.8	22.7
P4	14,576	22.4	5.1
P5	0	0.0	0.0
P6	13,313	4.6	4.6

Flow Cytometry Gating Strategy for Supplementary Figure S6f:
Myeloid Cells CD45+ F4/80+ CD11b+, From B16 Tumor Immune Cells in NRE1 fl/fl ± LysM-Cre mice.

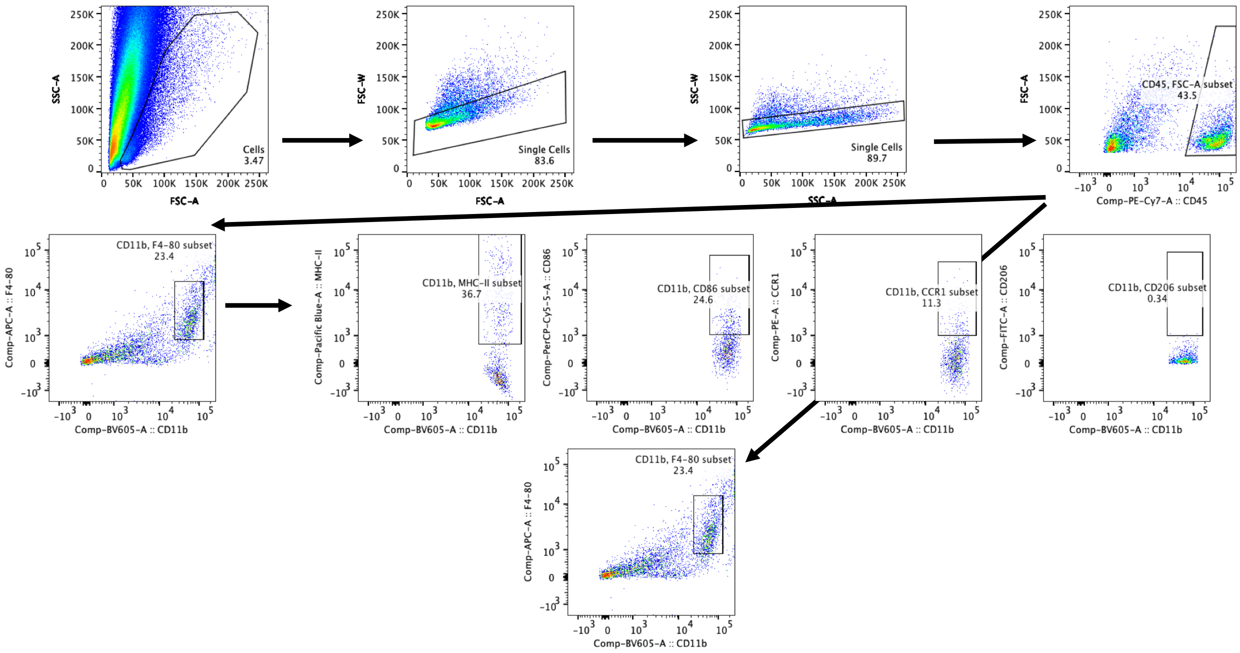

Supplementary Figure S6. Related to Figure 6. STAT1-inducible *Nampt* function in the hematopoietic lineage during malignancy. (A) Relative number of cells (percent total) from each of the identified cell types using the Seurat analysis of scRNAseq from B16F10-ova melanoma tumors in WT or NRE1 KO mice in Fig. 6B. Populations that were analyzed further are emphasized. **(B)** Cell frequencies for cell type populations from WT or NRE1 KO mice analyzed in Fig. 6C and 6D. **(C)** Relative expression levels from scRNAseq for characteristic identifying genes discriminating between dendritic cell types cDC1-like (migratory) as opposed to cDC2-like, or **(D)** cDC2-like as opposed to cDC1-like marker genes. **(E)** MTORC1 Signaling GSEA signatures as in Figure 6D for additional indicated myeloid cell cluster types from scRNAseq, with positive enrichment of gene sets in wild-type on the left of each plot relative to NRE1 KO on the right. NES: Normalized Enrichment Score, p_{adj}: Adjusted p-value:two tailed, corrected for multiple comparisons using Benjamini-Hochberg method. **(F)** Flow cytometric analysis of relative percentages of macrophages of indicated genotypes in B16F10-ova tumors. Flow cytometry gating strategy provided in Source Data file in Supplementary Information. **(G)** MC-38-ova COAD cell line flank tumor size (See Methods) growth rate in WT or LysM-Cre NRE1 fl/fl (NRE1-KO) mice. **(H)** *Nampt* expression in all scRNAseq cluster lymphoid and myeloid cell types from WT vs. NRE1 KO mice. Plots show the distribution of gene expression in individual cells, and the red dot denotes average expression across the cells within the sample. Crossbars, boxes, and whiskers represent median, interquartile range, and total range of *Nampt* expression, respectively. Outlined cell types are those from Figure 6C for comparison. For **(F, G)**, data are the average of N = 7 or 9 biological replicates. For (H), data are the average of N = 3 biological replicates. P values were determined by a two-tailed unpaired t-test: *p<.05, **p<.005, ***p<.0005, ****p<.00005. Error bars represent SEM. **(I)** Flow Cytometry Gating Strategies for Figure 6b Sorting, and Supplementary Figure S6f. Myeloid Cells CD45+ F4/80+ CD11b+ from B16 Tumor Immune Cells in NRE1 fl/fl ± LysM-Cre mice.

Supplementary Figure S7.

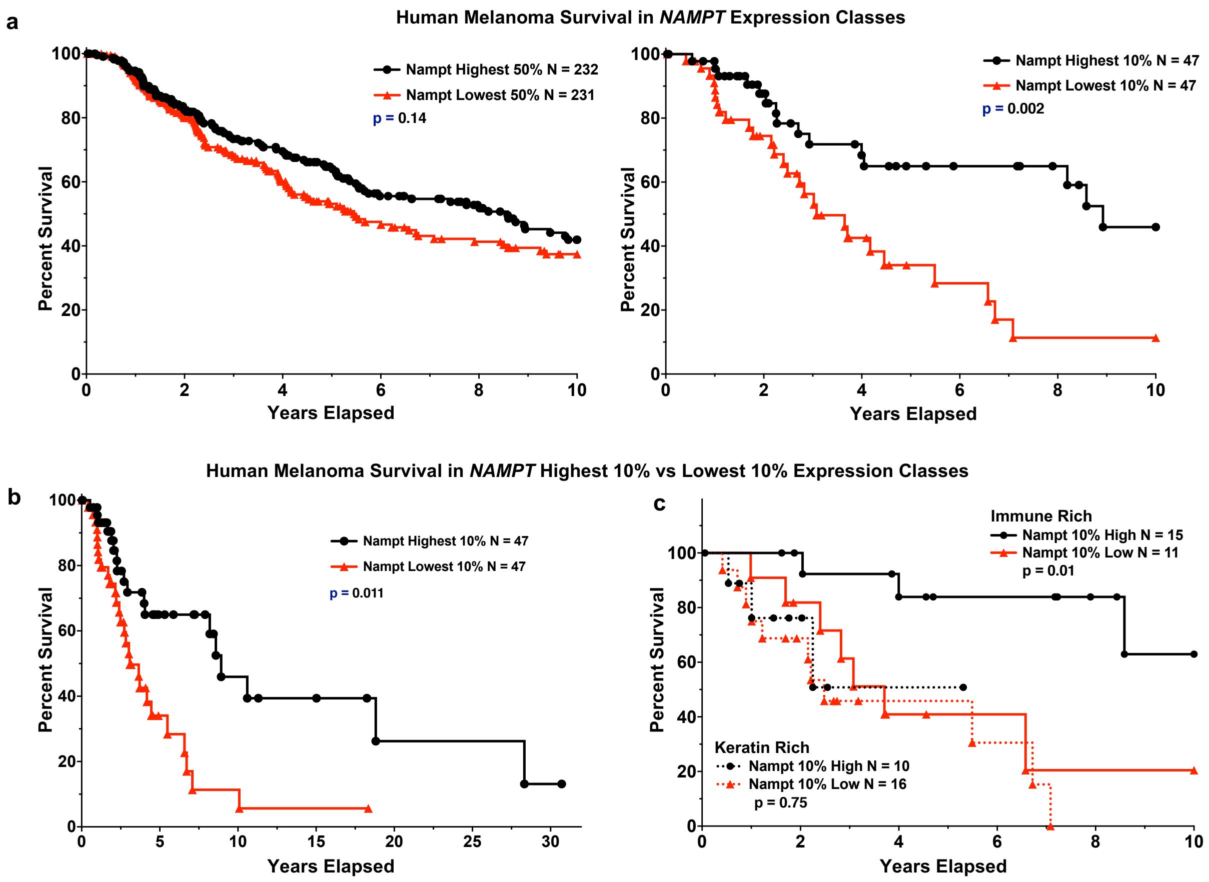

Supplementary Figure S7. Related to Figure 7. Immune cell *NAMPT* expression in human immune cells, and correlation with melanoma disease inflammation and outcome. (A) Kaplan-Meier curves of TCGA ten-year human melanoma patient survival data comparing median-divided *NAMPT* expression, that is, from patients with tumors expressing the highest vs. lowest 50% of *NAMPT* expression (left plot) to patients with tumors expressing the highest vs. lowest 10% of *NAMPT* expression levels (right plot). **(B)** Survival curves of full TCGA 30+ year melanoma patient survival data from patients with tumors expressing the highest vs. lowest 10% of *NAMPT* expression levels. **(C)** Ten-year survival data were further stratified with respect to both the highest vs. lowest 10% of *NAMPT* expression levels and tumors with high vs. low immune infiltration, as in Fig. 7A. P values are reported in the figure panels for the difference between high and low *NAMPT* expression plots for either the keratin signature or immune signature-rich samples, and are from the log-rank (Mantel-Cox) test.

Supplementary Table S1: Oligos used in this study.

RT-qPCR Primers			
Mouse			
ActB	F	o1097	GGCTGTATTCCCCTCCATCG
	R	o1098	CCAGTTGGTAACAATGCCATGT
Arg1	F	o629	TTGGGTGGATGCTCACACTG
	R	o630	TTGCCCATGCAGATTCCC
Ccr1	F	o1215	ACTGCTGTAAGAGCCTTTGGG
	R	o1216	AGCACCAGAATCACTAGGACA
Cd44	F	o1407	GGACTTTGCCTCTTGCAGTTGAGC
	R	o1408	TTTCTCCACATGGAATACACCTGCG
Cxcl10	F	o1101	TTGGGCATCATCTTCCTGGAG
	R	o1102	GAGGTCTTTGAGGGATTTGTA
Hif1α	F	o180	ACCTTCATCGGAAACTCCAAAG
	R	o181	ACTGTTAGGCTCAGGTGAACT
Hk2	F	o1439	CCTCAGCTGGTGAGCCATC
	R	o1440	ACTGGTCAACCTTCTGCACT
Il12b	F	o1258	TGGTTTGCCATCGTTTTGCTG
	R	o1259	ACAGGTGAGGTTCACTGTTTCT
Il6	F	o85	TAGTCCTTCCTACCCCAATTTCC
	R	o86	TTGGTCCTTAGCCACTCCTTC
LdhA	F	o1441	CGTGCACTAGCGGTCTCAAA
	R	o1442	GGAGATCCATCATCTCGCCC
Mir155hg (BIC)	F	o1038	TCCTCATGAAACCAGCTCATCTG
	R	o1040	AGCAGCCAGAGGATAAGGGAT
RpL32	F	o1332	ATCAGGCACCAGTCAGACC
	R	o1333	TTGAACCTTCTCCGCACCC
Nampt	F	o631	AGTGGCCACAAATTCCAGAG
	R	o632	CAATTCCCGCCACAGTATCT
Nos2	F	o1318	CAGCTGGGCTGTACAAACCTT
	R	o1319	CATTGGAAGTGAAGCGGTTCG
Pdk1	F	o1445	GGGCCAGGTGGACTTCTATG
	R	o1446	TGGATATACCAACTTTGCACCAG
Stat1	F	o661	TACGGAAAAGCAAGCGTAATCT
	R	o662	TGCACATGACTTGATCCTTCAC
TNFα	F	o1435	CTGAACTTCGGGGTGATCGG
	R	o1436	GCTACGACGTGGGCTACAG
Nampt NRE1	F	o1207	AACAGCCTGCTTTGGAGAAA
	R	o1208	ACATGCATGGAGGCAACATA

**RT-qPCR Primers**			
Human			
Hs NAMPT	F	o1055	TAAAAGCTGTTCCTGAGGGCT
	R	o1056	AGAATTTGTGGCCACTGTGATTG
Hs CXCL10	F	o1099	GTGGCATTCAAGGAGTACCTC
	R	o1100	TGATGGCCTTCGATTCTGGATT
Hs ACTB	F	o1071	GTCACCAACTGGGACGACAT
	R	o1072	GTACATGGCTGGGGTGTTGA
**Chip-qPCR Primers**			
Nampt -3700	F	o1139	CTTGGCGTTGTCTGCATGATGCT
	R	o1140	ATCCCAACCAAAGGCTACATTGACTG
Nampt +925	F	o1083	GGGGAGAGTAAAATGGCACA
	R	o1084	GCGGGCATTTCTTCATCA
Nampt +2600	F	o1149	GGCACATCTCCTACCCAGCTTT
	R	o1150	AGTCCCGAATGCAATCCAACC
Nampt +4000	F	o1153	CTGTGGCATTGAATATGTTAGCCC
	R	o1154	ATCACCAAAGGCAACTGGTACT
Nampt +5100	F	o1217	TTCAAGGAACGATTACTAGTAAGGTTG
	R	o1218	AGATCTTGACTCCTGTTAATTTGTCCA
Cxcl10-E2	F	o1105	GCCTCTGCTTCTGAGCTTCTTCTAAG
	R	o1106	GGCACATTTGCTTCGCTAGTATTTATTC
Gene Desert XVII	F	o1107	TCGACAGGCTGACCAATCCCA
	R	o1108	CCGGCAAGGAAAACGCAACAC
RpL32 ORF	F	o53	AAGCGAAACTGGCGGAAAC
	R	o54	TAACCGATGTTGGGCATCAG
**Genotyping Primers**			
NRE1 Left loxP (5’)	F	o1201	GTTAGGTAACAGCCCCTGGCTCG
	R	o1202	GTGCAAGAGTCAACTCAATAGGCAC
NRE1 Right loxP (3’)	F	o1203	CATAGGTTAACAGTTCTCTTTCATGGT
	R	o1204	ATGTGTATGAGTGTTACCACATTCATG
NRE1 5' CRISPR Site	F	o899	GACAGGGAGGACTGGATGAG
	R	o836	CCTCTCTTGGCTAAGGTGCAT
NRE1 3' CRISPR Site	F	o847	TAACATTGAAGCGGGGTCTC
	R	o900	AACAAACAAACAGCCCAAGG

CRISPR-Cas9 sgRNA encoding oligos	
NRE1 5' CRISPR	GGACTACTAAACTCCGGTCA
NRE1 3' CRISPR	GGAAATGCAACCCCACCGAG
SBS 5' CRISPR	ACTACGTGATTTCTGTGAAA
SBS 3' CRISPR	GCTTTCACGGAGGTTCGACG
Stat1 CRISPR 1	GGTACTGTCTGATTTCCATG
Stat1 CRISPR 2	GAGGAGGTCATGGAAGCGGA

Supplementary Table 2: Gene Symbols in this study.

Gene Symbol	Full Name	Aliases
Arg1	arginase, liver	Arg, Arg-1, PG, PGIF
Ccr1	chemokine (C-C motif) receptor 1	Cmkbr, Cmkbr1, Mip-1a-R
Cd44	CD44 antigen	HERM, HERMES, Ly-2, Ly-24, Pgp, Pgp-1
Gapdh	glyceraldehyde-3-phosphate dehydrogenase	Ga, Gapd
Hif1a	hypoxia inducible factor 1	HIF-1, HIF-1-alpha, HIF1, HIF1-alpha, HIF1alpha, MO, MOP1, bHLHe7, bHLHe78
Hk2	hexokinase 2	HK, HKII
Il12b	interleukin 12b	Il-1, Il-12, Il-12b, Il-12p40, Il12p40, p40
Il6	interleukin 6	Il, Il-6
LdhA	lactate dehydrogenase A	LDH, Ldh-, Ldh1, Ldhm, l7, l7R2
Jak1, 2	Janus kinase 1, 2	
Lyz2	lysozyme 2	LysM; L; Ly; Lz; Lys; Lzm; Lzp; Lyzs; Lzm-; Lyzf2; Lzm-s1
MHC II	histocompatibility-2, MHC	H-2, MHC-, MHC-II
Mir155hg	Mir155 host gene	Bi, Bic
Nampt	nicotinamide phosphoribosyltransferase	NAmPRTase, Pbef, Pbef1, Vi, Visfatin
Nos2	nitric oxide synthase 2	MAC-NOS, N, NOS-II, No, Nos-2a, i-NOS, iNOS, Nos2
Pdk1	pyruvate dehydrogenase kinase, isoenzyme 1	
Stat1	signal transducer and activator of transcription 1	
Tnfa	tumor necrosis factor	TNF-, TNF-a, TNF-alpha, TNFSF2, TNFalpha, Tna, Tnfs, Tnfsf1a, Tnlg1f, Tnf
