# Supplementary material for: A Stat1 bound enhancer promotes Nampt expression and function within tumor associated macrophages
Source: Nat Commun. 2021 May 11;12:2620. doi: 10.1038/s41467-021-22923-5 (PMC8113251; doi:10.1038/s41467-021-22923-5)
Supplement: Supplementary file 2 — Descriptions of Additional Supplementary Files [file 41467_2021_22923_MOESM2_ESM.pdf]

Descriptions of Additional Supplementary Files

Supplementary Data 1
Description: scRNASeq Marker Gene Expression for Tumor Immune Cell Cluster Assignment. A full description of the marker genes used to assign clusters, including expression level, frequency, and significance.

Supplementary Data 2
Description: GO Analysis of Differentially expressed genes in WT scRNASeq Clusters.

Supplementary Data 3
Description: GO Analysis of Differentially expressed genes in NRE1-KO scRNASeq Clusters.
