# Supplementary material for: A Stat1 bound enhancer promotes Nampt expression and function within tumor associated macrophages
Source: Nat Commun. 2021 May 11;12:2620. doi: 10.1038/s41467-021-22923-5 (PMC8113251; doi:10.1038/s41467-021-22923-5)
Supplement: Supplementary file 6 — Reporting Summary [file 41467_2021_22923_MOESM6_ESM.pdf]

nature research

Corresponding author(s): Warren P. Voth
Ryan M. O'Connell

Last updated by author(s): Mar 18, 2021

Reporting Summary

Nature Research wishes to improve the reproducibility of the work that we publish. This form provides structure for consistency and transparency in reporting. For further information on Nature Research policies, see our Editorial Policies and the Editorial Policy Checklist.

Statistics

For all statistical analyses, confirm that the following items are present in the figure legend, table legend, main text, or Methods section.

n/a	Confirmed	
☐	☒	The exact sample size (*n*) for each experimental group/condition, given as a discrete number and unit of measurement
☐	☒	A statement on whether measurements were taken from distinct samples or whether the same sample was measured repeatedly
☐	☒	The statistical test(s) used AND whether they are one- or two-sided<br>*Only common tests should be described solely by name; describe more complex techniques in the Methods section.*
☒	☐	A description of all covariates tested
☒	☐	A description of any assumptions or corrections, such as tests of normality and adjustment for multiple comparisons
☐	☒	A full description of the statistical parameters including central tendency (e.g. means) or other basic estimates (e.g. regression coefficient) AND variation (e.g. standard deviation) or associated estimates of uncertainty (e.g. confidence intervals)
☐	☒	For null hypothesis testing, the test statistic (e.g. *F*, *t*, *r*) with confidence intervals, effect sizes, degrees of freedom and *P* value noted<br>*Give P values as exact values whenever suitable.*
☒	☐	For Bayesian analysis, information on the choice of priors and Markov chain Monte Carlo settings
☒	☐	For hierarchical and complex designs, identification of the appropriate level for tests and full reporting of outcomes
☐	☒	Estimates of effect sizes (e.g. Cohen's *d*, Pearson's *r*), indicating how they were calculated

Our web collection on statistics for biologists contains articles on many of the points above.

Software and code

Policy information about availability of computer code

Data collection	No specialized software was used for data collection in the laboratory studies. Published TCGA data was downloaded using TCGABiolinks (v2.16.4) R package. TCGA RNAseq data were processed using established pipelines including EdgeR (v3.30.3) and limma (v3.44.3) in R environment.
Data analysis	scRNAseq data was analyzed using Seurat R package (v3.1.5) (Butler et al, 2018). Computer-aided annotation of single-cell clusters was performed using CIPR-Shiny (v0.1.0) algorithm we described previously (Ekiz HA, JCI Insight, 2019). This software is available at (https://aekiz.shinyapps.io/CIPR/). The source code for CIPR is found at (https://github.com/atakanekiz/CIPR-Shiny). Gene set enrichment analyses were performed using fgsea R package (vl.14.0) using gene lists ranked by signal-to-noise metric as described in (Subramanian et al, 2005). Other statistical analyses in the study were performed using Graph Pad Prism (v8.4.2). Gel and Western analysis used Biorad GelDoc XR+ and ImageLab 6.1.

For manuscripts utilizing custom algorithms or software that are central to the research but not yet described in published literature, software must be made available to editors and reviewers. We strongly encourage code deposition in a community repository (e.g. GitHub). See the Nature Research guidelines for submitting code & software for further information.

Data

Policy information about availability of data

All manuscripts must include a data availability statement. This statement should provide the following information, where applicable:

- Accession codes, unique identifiers, or web links for publicly available datasets
- A list of figures that have associated raw data
- A description of any restrictions on data availability

All RNA sequencing data have been deposited in Gene Expression Omnibus under GEO: GSE113836, GSE113837, GSE123822, GSE135814. The figures which have

associated deposited raw data are: Fig. 1, Fig. 3, Fig. 6, Fig. 52, Fig. 53, and Fig. 56. These datasets are freely available.

Field-specific reporting

Please select the one below that is the best fit for your research. If you are not sure, read the appropriate sections before making your selection.

- [x] Life sciences
- [ ] Behavioural & social sciences
- [ ] Ecological, evolutionary & environmental sciences

For a reference copy of the document with all sections, see nature.com/documents/nr-reporting-summary-flat.pdf

Life sciences study design

All studies must disclose on these points even when the disclosure is negative.

Sample size

Sample sizes were determined by pilot experiments to evaluate amplitude and variation of differences, and were increased if needed for replicated experiments presented in the manuscript, or were evaluated by the University of Utah Biostatistics core facility.

Data exclusions

No datapoints were excluded from the experimental data presented in this manuscript.

Replication

Reproducibility of experimental findings was verified by independently repeating experiments in their entirety at least two times, as described in the manuscript. With the incidental exception of some pilot experiments not reported in this manuscript, presented experiments all showed the same trends and in most cases, similar levels of significance. Lack of replication of pilot experiments was due to issues such as inadequate sample sizes, or the need for procedural improvements.

Randomization

Organisms and samples were randomly allocated into different experimental groups based on segregation of mouse and cell lines and genotyping to confirm their differences. Animals of the appropriate genotypes were then allocated into experimental groups described , as in the manuscript, as WT vs IFNgR deficient, or NREfl/fl vs. NREfl/fl bearing the appropriate Cre driver. These groups were also age and sex matched.
Any possible covariates were controlled for by using mice from separate parallel crosses, by using
littermate controls, controls bearing each of the genetic manipulations of the test animals or samples tested individually, and by testing for the desired genetic deletions in the final experimental samples that were analyzed.

Blinding

No interventional or observational treatment studies were performed.
Some experimental measurements were blinded by using separate researchers to analyze samples that were constructed and prepared by different experimenters and assigned non-descriptive numbers for the samples. Tumor mass measurements were accomplished by blinded researchers performing the dissection and weight measurements without information as to the experimental animal groups from which the samples were sourced. Otherwise, Blinding was not relevant to other aspects of this study because
the samples were intrinsically defined and identified during construction, preparation, and analysis.

Reporting for specific materials, systems and methods

We require information from authors about some types of materials, experimental systems and methods used in many studies. Here, indicate whether each material, system or method listed is relevant to your study. If you are not sure if a list item applies to your research, read the appropriate section before selecting a response.

Materials & experimental systems

n/a	Involved in the study	
[ ]	[x]	Antibodies
[ ]	[x]	Eukaryotic cell lines
[x]	[ ]	Palaeontology and archaeology
[ ]	[x]	Animals and other organisms
[ ]	[ ]	Human research participants
[ ]	[x]	Clinical data
[x]	[ ]	Dual use research of concern

Methods

n/a	Involved in the study	
[x]	[ ]	ChIP-seq
[ ]	[x]	Flow cytometry
[x]	[ ]	MRI-based neuroimaging

Antibodies

Antibodies used

NAMPT (Bethyl Laboratories PBEF, A300-372A) 1:1000 dilution
STAT1 (Santa Cruz sc-346, sc-529) 1:1000 dilution
ACTB (Santa Cruz sc-47778) 1:2000 dilution
CD45 APC (30-F11, BioLegend 103112) 1:400 dilution
CCR1 PE (Fisher Scientific FAB5986P025) 1:200 dilution
CD11b-BV605 (M1/70, BioLegend 101257) 1:200 dilution
F4/80 PE (BM8, BioLegend 123119) 1:200 dilution

	F4/80 APC (BM8, BioLegend 123115) 1:200 dilution<br>CD16/32 Blocking antibody (Clone 93, BioLegend 101301) 1:200 dilution<br>CD45 PE-cy7 (BioLegend 103113) 1:400 dilution<br>CD45 PB (BioLegend 103125) 1:400 dilution<br>Ly6c FITC (BioLegend 128005)1:200 dilution<br>MHCII PB BioLegend 107619) 1:200 dilution<br>Gr1 PE BioLegend 108407) 1:2000 dilution<br>CD40 FITC BioLegend 124607)1:200 dilution<br>CD86 Percp-cy5.5 (BioLegend 105027)1:200 dilution<br>CD11b Percp-cy5.5 (BioLegend 101227)1:200 dilution<br>CD11b APC (BioLegend 101211) 1:200 dilution
Validation	NAMPT (Bethyl Laboratories PBEF, A300-372A), validation by manufacturer for Western Blot<br>STAT1 (Santa Cruz sc-346, sc-529), validation by manufacturer, and data in this manuscript using genetically deleted cell lines showing no cross reactivity from these antibodies, for Western Blots and ChIP.<br>ACTB (Santa Cruz sc-47778), validation by manufacturer for Western Blot<br>CD45 APC (30-F11, BioLegend 103112), validation by manufacturer for Flow Cytometry of mouse cells<br>CCR1 PE (Fisher Scientific FAB5986P025), validation by manufacturer for Flow Cytometry of mouse cells<br>CD11b-BV605 (M1/70, BioLegend 101257), validation by manufacturer for Flow Cytometry of mouse cells<br>F4/80 (BM8, BioLegend 123110), validation by manufacturer for Flow Cytometry of mouse cells<br>CD16/32 Blocking antibody (Clone 93, BioLegend 101301), validation by manufacturer for Flow Cytometry of mouse cells<br>CD45 PE-cy7 (BioLegend 103113), validation by manufacturer for Flow Cytometry of mouse cells<br>CD45 PB (BioLegend 103125), validation by manufacturer for Flow Cytometry of mouse cells<br>Ly6c FITC (BioLegend 128005), validation by manufacturer for Flow Cytometry of mouse cells<br>MHCII PB BioLegend 107619), validation by manufacturer for Flow Cytometry of mouse cells<br>Gr1 PE BioLegend 108407), validation by manufacturer for Flow Cytometry of mouse cells<br>CD40 FITC BioLegend 124607), validation by manufacturer for Flow Cytometry of mouse cells<br>CD86 Percp-cy5.5 (BioLegend 105027), validation by manufacturer for Flow Cytometry of mouse cells<br>CD11b Percp-cy5.5 (BioLegend 101227), validation by manufacturer for Flow Cytometry of mouse cells<br>CD11b APC (BioLegend 101211), validation by manufacturer for Flow Cytometry of mouse cells

Eukaryotic cell lines

Policy information about cell lines

Cell line source(s)	RAW264.7: RAW 264.7 (ATCC #TIB-71) The RAW264.7 parental cell line has not been re-authenticated or subjected to mycoplasma testing in the past year. Cells were grown in DMEM with 10% FBS, penicillin and streptomycin at 37C with 5% CO2, then frozen in liquid nitrogen. For all experiments, cells were recovered from frozen aliquots before experimental manipulation and analysis.<br>B16F10: Described in:<br>Jeremy P. Snook, Ashleigh J. Soedel, H. Atakan Ekiz, Ryan M. O'Connell and Matthew A. Williams Cancer Immunol Res February 19 2020 DOI: 10.1158/2326-6066.CIR-19-0690:<br>Mouse B16-F10 cells were purchased from American Type Culture Collection (Manassas, VA) in 2014 and grown in DMEM with 10% FBS, penicillin and streptomycin at 37C with 5% CO2, then frozen in liquid nitrogen. For all experiments, cells were recovered from frozen aliquots and cultured for 1-2 weeks prior to inoculation of mice.<br>293-T: The 293T (ATCC CRL-3216) parental cell line has not been re-authenticated or subjected to mycoplasma testing in the past year. Cells were grown in DMEM with 10% FBS, penicillin and streptomycin at 37C with 5% CO2, then frozen in liquid nitrogen. For all experiments, cells were recovered from frozen aliquots before experimental manipulation and analysis.
Authentication	Cells lines were authenticated by gene expression under experimental conditions, the expression of fluorescent markers, and morphology.
Mycoplasma contamination	The cell lines have not been subjected to mycoplasma testing in the past year.
Commonly misidentified lines<br>(See ICLAC register)	*Name any commonly misidentified cell lines used in the study and provide a rationale for their use.*

Animals and other organisms

Policy information about studies involving animals; ARRIVE guidelines recommended for reporting animal research

Laboratory animals	Mus musculus Strain C57BL/6J, male or female, ages 8-11 weeks<br>Mus musculus Strain C57BL/6J NRE1 fl/fl, Biocytogen, male or female, ages 8-11 weeks
Wild animals	none
Field-collected samples	none
Ethics oversight	All animal laboratory work was approved by the University of Utah Health IACUC and the Comparative Medicine Center.

Note that full information on the approval of the study protocol must also be provided in the manuscript.

Human research participants

Policy information about studies involving human research participants

Population characteristics	*Describe the covariate-relevant population characteristics of the human research participants (e.g. age, gender, genotypic information, past and current diagnosis and treatment categories). If you filled out the behavioural & social sciences study design questions and have nothing to add here, write "See above."*
Recruitment	*Describe how participants were recruited. Outline any potential self-selection bias or other biases that may be present and how these are likely to impact results.*
Ethics oversight	*Identify the organization(s) that approved the study protocol.*

Note that full information on the approval of the study protocol must also be provided in the manuscript.

Clinical data

Policy information about clinical studies

All manuscripts should comply with the ICMJEguidelines for publication of clinical research and a completedCONSORT checklist must be included with all submissions.

Clinical trial registration	*Provide the trial registration number from ClinicalTrials.gov or an equivalent agency.*
Study protocol	*Note where the full trial protocol can be accessed OR if not available, explain why.*
Data collection	*Describe the settings and locales of data collection, noting the time periods of recruitment and data collection.*
Outcomes	*Describe how you pre-defined primary and secondary outcome measures and how you assessed these measures.*

Flow Cytometry

Plots

Confirm that:

- [x] The axis labels state the marker and fluorochrome used (e.g. CD4-FITC).
- [x] The axis scales are clearly visible. Include numbers along axes only for bottom left plot of group (a 'group' is an analysis of identical markers).
- [x] All plots are contour plots with outliers or pseudocolor plots.
- [x] A numerical value for number of cells or percentage (with statistics) is provided.

Methodology

Sample preparation	After mechanical disruption of tumor tissue, tumor cells were placed on an orbital shaker in Accumax (Innovative Cell Technologies), incubated for thirty minutes at room temperature, and followed by red blood cell lysis were filtered through a 0.45-micron filter to obtain a single cell suspension. Cells were stained with a combination of fluorophore-conjugated antibodies in HBSS supplemented with 10% BSA, pyruvate, EDTA, and HEPES.
Instrument	Becton Dickinson LSR2 Fortessa
Software	FlowJo 10, Becton Dickinson
Cell population abundance	Relative abundance of TAMs in harvested tumors ranged from 30% to 50% of CD45+ leukocytes. Purity was ensured by F4/80 and CD11b positivity above 2x10^3 fluorescence units for each.<br>Relative abundance of CD45+ TILS in purification for scRNASeq was 36.4% (6.3% of Total) for WT, 22.4% (5.1% of Total) for NRE-ko. Post-sorting purity was 96%.
Gating strategy	FSC-Width/SSC-Area data was used for gating out debris (FSC-low, SSC-low), with thresholds of 1x10^4 and 4x10^4 respectively. Cell doublets were removed by gating out FSC(width)-hi and SSC(width)-hi events. Subsequently, positive gates were determined using unstained sample (or fluorescent-minus-one (FMO) controls, where applicable).

- [x] Tick this box to confirm that a figure exemplifying the gating strategy is provided in the Supplementary Information.
